# Spatial cell-cell communication inference based on cell-spot-ligand-receptor heterogeneous graphs

**DOI:** 10.1371/journal.pcbi.1014835

**Published:** 2026-09-28

**Authors:** Xiong Li, Dongding Wu, Yuejin Zhang, Min Chen, Chong Liu

**Affiliations:** 1 School of Information and Software Engineering, East China Jiaotong University, Nanchang, Jiangxi, China; 2 College of Computer Science and Engineering, Hunan Institute of Technology, Hengyang, Hunan, China; University of California Irvine, UNITED STATES OF AMERICA

## Abstract

Cell-cell communication is essential for tissue homeostasis and disease-associated microenvironment remodeling. Spatial transcriptomics enables the study of communication events *in situ*, but single-cell-resolution inference from spot-based data remains challenging because of uncertain cell-to-spot mapping, limited use of local spatial information, and false positives from ligand-receptor co-expression. Existing methods increasingly emphasize spatial proximity in cell-cell communication inference, while integrating biologically informative signals remains important for improving the interpretability of candidate communication events. Here, we present SpaHCC, a heterogeneous graph learning framework that integrates single-cell transcriptomics, spatial transcriptomics, and prior ligand-receptor knowledge for spatial cell-cell communication inference. By jointly leveraging molecular features, spatial neighborhood information, and cellular functional states, SpaHCC prioritizes candidate communication events and supports their biological contextualization through pathway, transcription factor, and receptor-side response analyses. Benchmark and spatial consistency analyses showed that SpaHCC recovered spatially coherent candidate communication signals. In Alzheimer’s disease data, SpaHCC identified recurrent excitatory neuron-associated candidate patterns, including NRXN- and FLRT2-related modules associated with synaptic remodeling and inflammation-related transcriptional programs. Cross-patient analyses showed recurrence of selected AD-associated candidate patterns, while representative cSCC and PDAC analyses illustrated the spatial interpretability of the framework in additional disease contexts. Overall, SpaHCC provides an effective and interpretable framework for spatially resolved cell-cell communication analysis in complex diseases.

## Author summary

Cells in tissues constantly exchange signals, and these interactions help shape both normal tissue function and disease. Recent studies have increasingly used spatial transcriptomics to examine how the positions of cells influence potential communication patterns within tissues. However, spatial proximity alone is often not sufficient to understand whether two cells are likely to communicate in a biologically meaningful way, because being close in space does not necessarily mean that they are truly engaged in meaningful signaling interactions. We developed SpaHCC to combine spatial information with ligand-receptor evidence and cellular state information, so that candidate communication events can be interpreted in a more biologically informed manner. When applied to Alzheimer’s disease data, SpaHCC highlighted candidate communication patterns centered on excitatory neurons, which were associated with synaptic and inflammation-related programs. Our work provides a computational framework for studying not only where cell-cell communication may occur, but also what it may mean biologically in complex diseases.

## Introduction

Cell-cell communication (CCC) is a fundamental mechanism that maintains tissue homeostasis, regulates neural activity, mediates immune responses, and drives disease progression. Many intercellular signaling events are mediated by ligand-receptor interactions between sender and receiver cells, making systematic characterization of these interactions important for understanding how cellular communities are reorganized in complex diseases [1–3]. In recent years, single-cell transcriptomics has enabled large-scale inference of putative communication networks based on cellular heterogeneity and ligand-receptor co-expression patterns. However, because dissociated single-cell data do not preserve native spatial context, these approaches often cannot determine whether candidate communication events occur between cells that are spatially proximal or physically reachable *in situ*.

Spatial transcriptomics preserves tissue architecture and local neighborhood relationships, creating new opportunities to study cell-cell communication *in situ*. In recent years, a growing number of methods have incorporated spatial information into communication inference. For example, Giotto, COMMOT, SpaTalk, DeepTalk, Spacia, and CellNEST improve the spatial plausibility of inferred interactions through neighborhood modeling, optimal transport, knowledge-graph modeling, graph learning, multiple-instance learning, or local signal propagation, respectively [4–9]. In addition, some methods have explored strategies such as multi-view graph learning to further characterize spatial communication patterns [10]. Recent deep-learning methods have also advanced related spatial and single-cell omics tasks, including spatial gene expression prediction, spatial transcriptomics imputation, and cell-specific gene regulatory network inference [11–13]. Recent reviews have further emphasized that next-generation CCC methods are increasingly moving beyond simple ligand-receptor co-expression toward frameworks that jointly model spatial organization, intracellular signaling, and multimodal integration [14,15]. Despite these advances, many existing approaches emphasize spatial distance, neighborhood structure, or signal propagation, while the integration of receptor-side cellular states and downstream functional context remains an important direction for spatial CCC analysis.

To address these challenges, we developed SpaHCC, a heterogeneous graph learning framework that integrates single-cell transcriptomics, spatial transcriptomics, and prior ligand–receptor knowledge to infer spatially supported candidate cell–cell communication events. SpaHCC jointly models cells, spatial spots, and ligand–receptor pairs in a unified heterogeneous graph, and characterizes sender cell–ligand–receptor–receiver cell triads by integrating molecular evidence, spatial neighborhood information, and cellular functional states. Rather than discovering new ligand–receptor pairs de novo, SpaHCC provides spatial and directional resolution for candidate communication events within a curated ligand–receptor universe. In Alzheimer’s disease data, SpaHCC identified recurrent excitatory neuron-associated candidate communication patterns, including NRXN- and FLRT2-related modules associated with synaptic remodeling and inflammation-related transcriptional programs. Representative external analyses in cSCC and PDAC further illustrate the spatial interpretability of the framework across disease contexts.

## Results

### SpaHCC framework and workflow for single-cell-resolution spatial cell–cell communication inference

To infer cell–cell communication at single-cell resolution in a spatial context, we developed SpaHCC, a tripartite heterogeneous graph framework that integrates single-cell transcriptomics, spatial transcriptomics, and prior ligand–receptor information (Fig 1). First, single cells were softly mapped to spatial spots using Tangram, and cell-to-spot edges incorporating both probabilistic and geometric information were explicitly constructed. Pathway activity and transcription factor activity at the single-cell level were then aggregated to the spot level, thereby establishing consistent functional representations across both the single-cell and spatial scales [16–19]. SpaHCC jointly models cells, spatial spots, and ligand–receptor (LR) pairs as three types of nodes in a heterogeneous graph. In this framework, LR nodes are constructed from a curated ligand–receptor candidate universe compiled from prior LR resources, including CellPhoneDB and CellChat, and are further characterized by their subunit composition and functional annotations. Based on these three node types, the model further establishes multiple types of edges to describe cell-to-spot correspondence, molecular associations on the sender and receiver sides, support from the local spatial environment for ligand–receptor signaling, and neighborhood relationships among spatial spots (Fig 1A and 1B).

**Fig 1:**
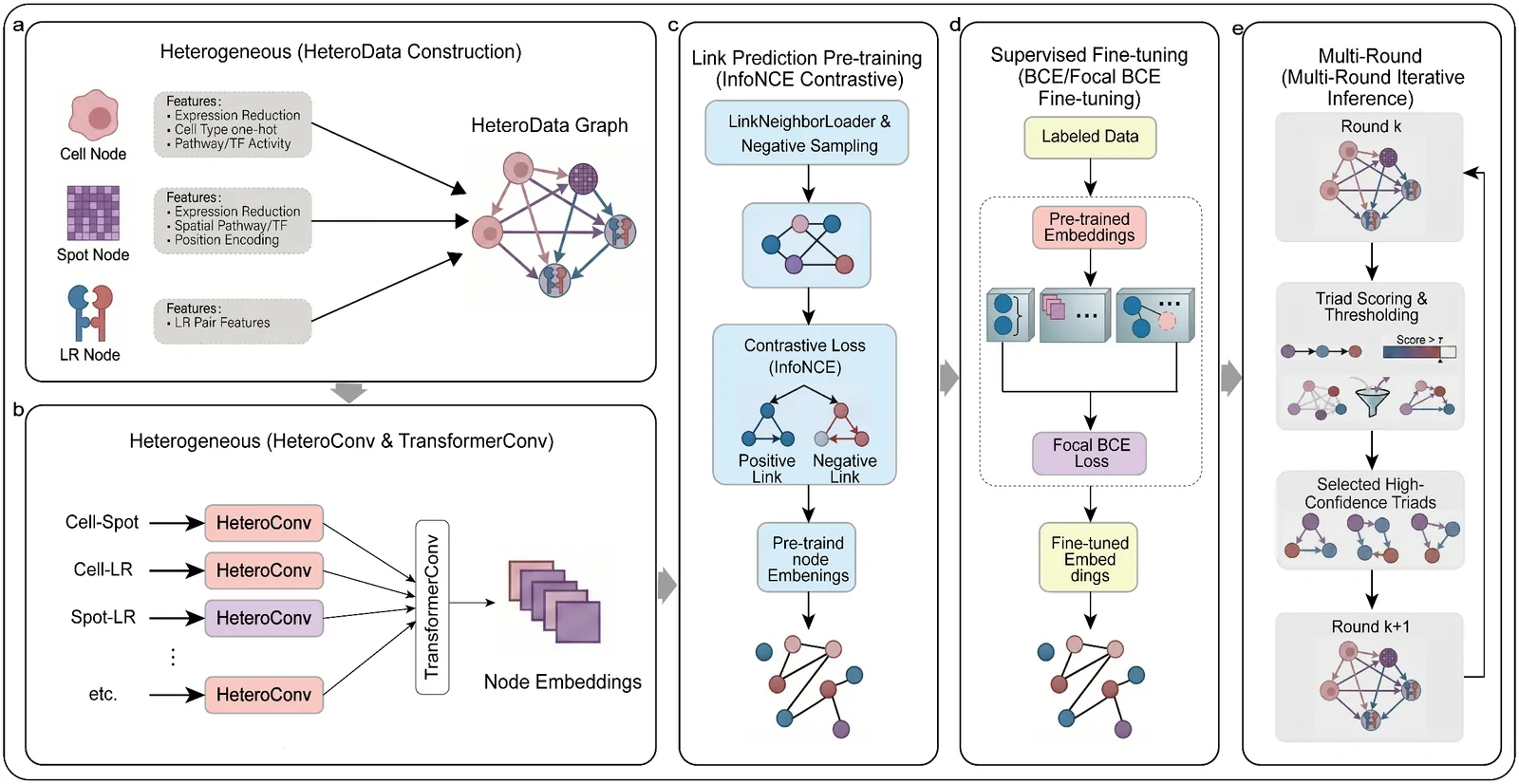
Overview of SpaHCC for single-cell-resolution spatial cell-cell communication inference in spatial transcriptomics. **(A)** Construction of a tripartite heterogeneous graph from single-cell transcriptomics, spatial transcriptomics, and prior ligand-receptor (LR) knowledge. **(B)** Joint representation learning of cell, spot, and LR nodes using a heterogeneous graph encoder. **(C)** Self-supervised contrastive pretraining on heterogeneous graph mini-batches. **(D)** Link prediction-based fine-tuning with online negative sampling. **(E)** Triad scoring and multi-round iterative refinement with spatial relay to prioritize high-confidence candidate sender cell–LR–receiver communication events.

On this basis, SpaHCC employs an encoder that combines heterogeneous graph convolution with Transformer-based attention to jointly learn relational information from different edge types in the graph, and obtains representations for cells, spatial spots, and ligand–receptor pairs through a pretraining-then-fine-tuning strategy (Fig 1C and 1D). Previous studies have shown that this modeling and training scheme is both feasible and effective [20,21]. Finally, SpaHCC scores candidate communication triads composed of a sender cell, a ligand–receptor pair, and a receiver cell, and further incorporates spatial relay information on the spot graph together with multi-round iterative screening. By progressively refining the candidate space using high-scoring results from the previous round, the model focuses on a more discriminative set of candidates. This iterative procedure identifies and refines single-cell candidate communication events supported by molecular evidence, spatial plausibility, and cross-round stability (Fig 1E) [7,21].

### Inferred intercellular communication patterns and key ligand–receptor modules in Alzheimer’s disease

We compared communication differences between the Alzheimer’s disease (AD) group and the control group at the level of cell-type interactions. The results showed that these changes were not broadly distributed across all cell-type pairs, but were instead concentrated in a limited number of specific interaction directions (Fig 2A). Among them, communication between excitatory neurons showed the most prominent alteration. In addition, interactions directed from astrocytes, inhibitory neurons, and oligodendrocyte precursor cells toward excitatory neurons were also markedly changed (Fig 2B). These findings suggest that AD-associated communication remodeling is primarily concentrated in interactions involving excitatory neurons, while also being accompanied by coordinated changes across multiple neuron–glia interaction axes.

**Fig 2:**
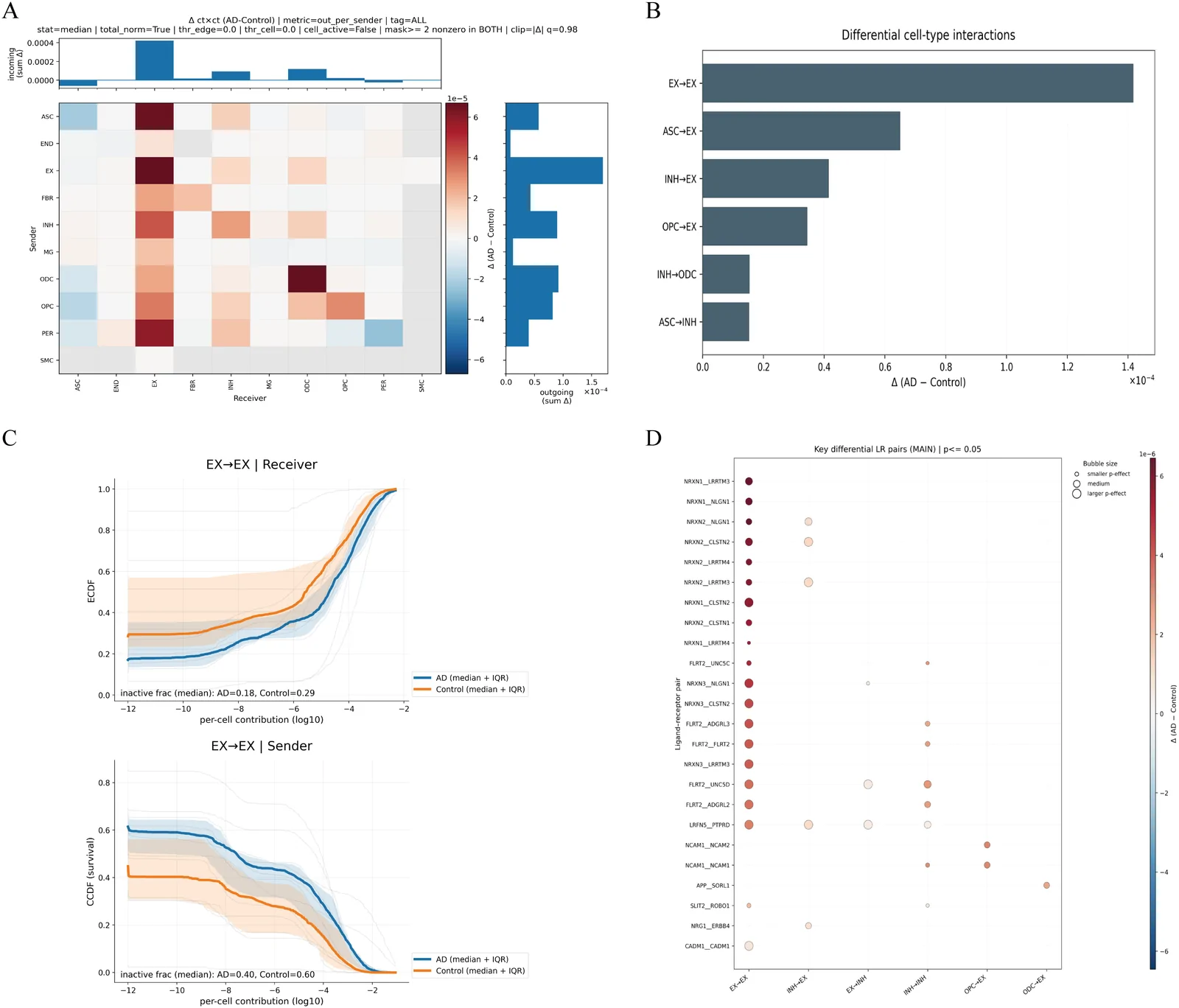
Multilevel characterization of differential spatial cell–cell communication in Alzheimer’s disease. **(A)** Heatmap showing differential cell-type interaction patterns between Alzheimer’s disease (AD) and control groups. **(B)** Bar plot summarizing the major differential interaction directions across cell-type pairs. **(C)** Changes in received and sent communication strengths for the EX→EX interaction axis at the single-cell level. **(D)** Representative differential ligand–receptor pairs and their associated functional modules underlying communication remodeling in AD.

Because the most pronounced communication changes were observed between excitatory neurons, we further examined the distribution of cell-level contributions within this interaction at single-cell resolution (Fig 2C). The results showed that, on both the sender and receiver sides, single-cell contributions were globally higher in the AD group, with fewer cells showing low contribution values. This indicates that more excitatory neurons participate in this interaction under the disease condition. Therefore, AD-associated communication changes are reflected not only in increased overall communication strength, but also in a redistribution of single-cell contributions. This pattern suggests that the observed enhancement is driven by broader cellular participation rather than by a small number of highly contributing cells.

At the ligand–receptor level, the representative candidate molecules were found to cluster into two major functional modules (Fig 2D). One module consisted of NRXN-related trans-synaptic adhesion interactions, including combinations such as NRXN–NLGN, NRXN–LRRTM, and NRXN–CLSTN. The other was an adhesion and axon-guidance module centered on FLRT2, including combinations such as FLRT2–ADGRL and FLRT2–UNC5. The former is mainly associated with synaptic recognition, synapse organization, and stabilization of neuronal connectivity [22,23], whereas the latter is closely related to cell adhesion, neural guidance, and connectivity remodeling [24]. In addition, LRFN5–PTPRD and CADM1–CADM1 were also identified among the representative candidate LR pairs, indicating that molecules involved in synaptic stability and plasticity also participate in this process [25,26]. Together, these findings suggest that altered communication involving excitatory neurons in AD is more likely to reflect coordinated remodeling across multiple molecular modules related to neuronal connectivity, rather than isolated abnormalities of individual molecules. The presence of a smaller number of candidate LR pairs in other cell-type interaction directions further suggests that this communication remodeling may extend to multiple neuron–glia interaction axes.

### Ground-truth benchmark, spatial consistency, and cross-method event prioritization

To assess SpaHCC using ground-truth communication labels, we evaluated it on the COMMOT simulated cell–cell communication benchmark. We selected seven representative simulation scenarios, with three simulated datasets included for each scenario, resulting in 21 simulated datasets in total. These datasets provide known sender–receiver–ligand–receptor labels, allowing direct evaluation of whether each method can recover labeled communication events. To reduce potential information leakage, we performed this benchmark under a leakage-controlled held-out setting, in which graph edges that directly encoded validation or test sender–receiver–LR labels were excluded from the training graph before model training, and AUROC was calculated only on the held-out test labels. Because the simulated ground-truth labels define sender–receiver–LR communication events rather than local spatial-neighborhood enrichment, we used a benchmark-specific SpaHCC configuration that retained spatial information in the encoder but excluded the explicit spatial terms from the final scoring step, referred to here as SpaHCC spatial-encoder-only. Under this leakage-controlled held-out setting, SpaHCC spatial-encoder-only achieved an average AUROC of 0.917, comparable to COMMOT (0.917) and higher than CellNEST (0.625), CytoSignal (0.799), Giotto expression-based scoring (0.873), and Giotto spatial scoring (0.438). The lower performance of CellNEST and Giotto spatial scoring is consistent with the possibility that strong spatial-neighborhood constraints are not fully aligned with the simulated ground-truth labels in this benchmark. We further performed a three-way spatial ablation comparing pure non-spatial SpaHCC (no spatial information in the encoder or final score), SpaHCC spatial-encoder-only (spatial encoder without explicit spatial scoring terms), and full-spatial SpaHCC (spatial encoder with the complete spatial score), which achieved mean AUROCs of 0.695, 0.917, and 0.523, respectively. These results indicate that non-spatial compatibility alone provides predictive signal, while spatial information in the encoder provides an additional contribution. The lower AUROC after restoring the explicit spatial terms indicates that these terms did not improve discrimination of the predefined labels in this benchmark, and their effect may therefore depend on the specific evaluation setting and target definition. Sample-level AUROC values for the two newly added variants are provided in S11 Table.

We further assessed whether SpaHCC could identify spatially enriched LR signals in the Alzheimer’s disease spatial transcriptomics data using the distance enrichment score (DES) [27]. DES measures whether high-scoring LR signals are enriched within local spatial neighborhoods and was therefore used here as a spatial enrichment and consistency metric rather than as the primary benchmark metric. SpaHCC showed a relatively high DES among the compared methods, with a mean DES of 0.48 ± 0.18, compared with COMMOT (0.43 ± 0.16) [5], Giotto (0.35 ± 0.12) [4], and CellNEST (0.33 ± 0.13) [9]. In contrast, CellChat [28], Scriabin [29], and CellCall [30] showed relatively lower DES values, and some methods had negative mean scores (Fig 3B). These results suggest that SpaHCC-inferred LR signals exhibit spatial enrichment in the AD tissue context. Representative spatial visualizations and related parameter-sensitivity analyses are provided in S1 and S2 Figs as complementary analyses.

**Fig 3:**
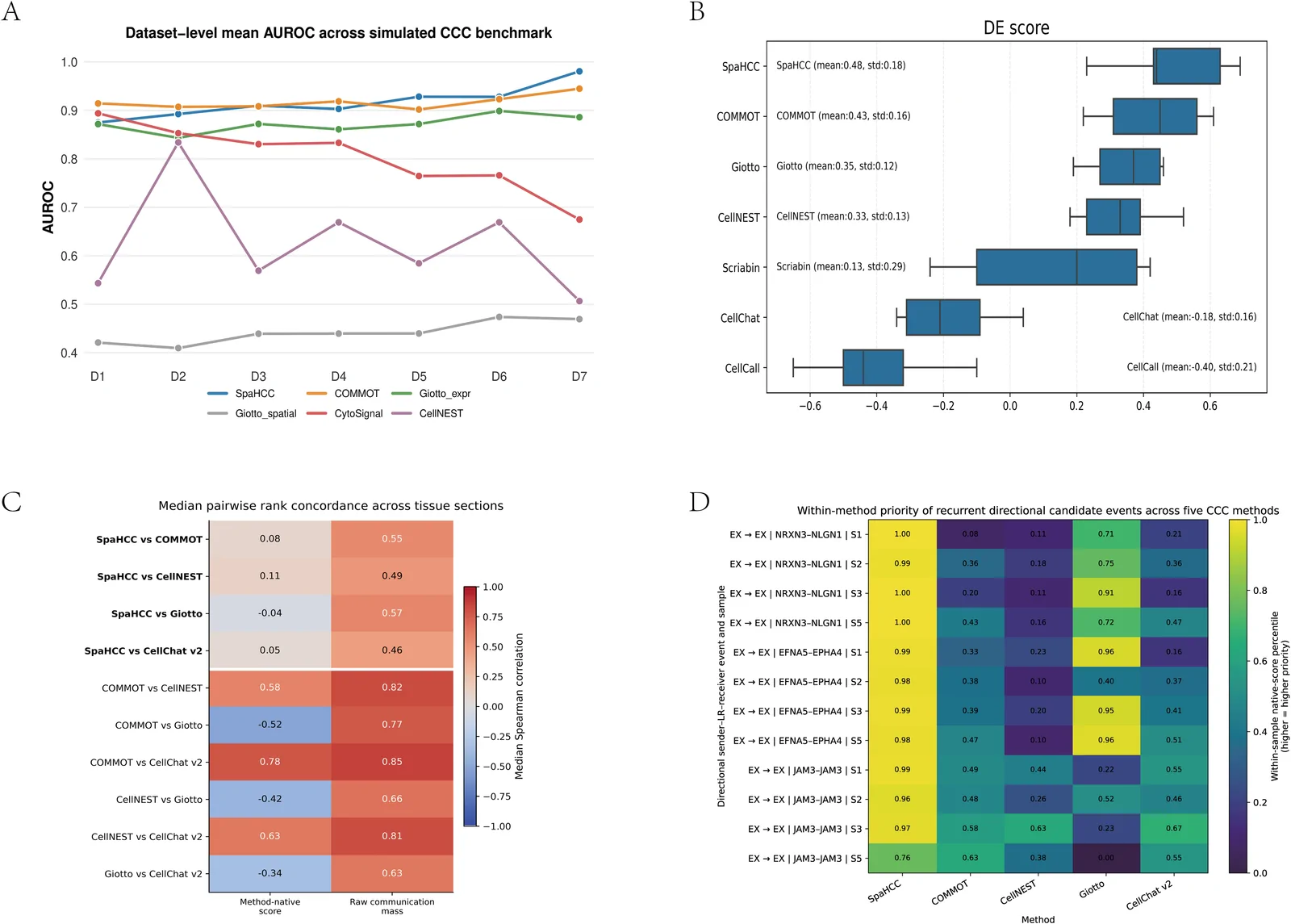
Ground-truth benchmark, spatial consistency, and cross-method prioritization of candidate communication events. **(A)** AUROC across COMMOT simulated cell–cell communication benchmark datasets. SpaHCC spatial-encoder-only achieved an average AUROC comparable to COMMOT and higher than CellNEST, CytoSignal, Giotto expression-based scoring, and Giotto spatial scoring under the matched evaluation setting. **(B)** Comparison of distance enrichment scores (DES) across methods in the Alzheimer’s disease dataset. DES was used as a spatial enrichment and consistency metric rather than as the primary benchmark metric. **(C)** Median pairwise Spearman rank concordance across tissue sections within the largest exact event universe shared by each method pair, shown for method-specific event-score rankings and communication mass. **(D)** Within-method percentiles of recurrent AD-associated directional candidate events in the strict all-five common event universe; higher percentiles indicate higher within-method priority.

To compare directional event prioritization across methods, we used two complementary harmonized event universes. Global rank concordance was evaluated within the largest exact sender–ligand–receptor–receiver event universe shared by each method pair, retaining only events with matched sender cell type, LR pair, and receiver cell type within the same section, whereas exact five-method candidate-event comparisons were performed in a stricter all-five common universe containing 5,637 matched events across five tissue sections. In the method-pair-specific shared universes, the median Spearman correlations between SpaHCC and COMMOT, CellNEST, Giotto, and CellChat v2 were 0.08, 0.11, −0.04, and 0.05, respectively, for method-specific event-score rankings. For comparison, we also evaluated communication-mass ordering, which summarizes lower-level spatial communication contributions for each matched event; its detailed definition and calculation are provided in S8 Table. The corresponding correlations based on communication-mass ordering were 0.55, 0.49, 0.57, and 0.46, respectively (Fig 3C). The corresponding analysis under the stricter all-five common-event universe is provided in S3 Fig A as a sensitivity analysis. Correlations based on communication-mass ordering were generally higher than those based on method-specific event-score rankings, whereas concordance in method-specific event prioritization was lower.

These differences were also evident for recurrent AD-associated candidate events (Fig 3D). EX→NRXN3–NLGN1→EX ranked 2/1,400, 21/1,500, 5/1,500, and 3/832 by SpaHCC across four evaluable sections, with a cross-section median percentile of 0.997 compared with 0.269 for the median across the four comparator methods. EX→EFNA5–EPHA4→EX and EX→JAM3–JAM3→EX showed similar recurrent prioritization patterns across sections. Summary statistics for these candidate events, together with complete five-method scores, exact ranks, and percentiles for EX→NRXN3–NLGN1→EX, are provided in S8 Table. The corresponding SpaHCC-minus-comparator percentile differences across evaluable sections are shown in S3 Fig B.

We next fixed the LR identity and compared alternative sender–receiver realizations associated with the same LR pair. Using a conservative scRNA-seq support score defined as the minimum of the ligand-sender AUC and receptor-receiver AUC, the cross-section median within-LR support percentile of the top-ranked realization was 0.891 for SpaHCC, compared with 0.711 for COMMOT, 0.650 for CellNEST, 0.614 for Giotto, and 0.888 for CellChat v2. SpaHCC showed higher conservative support than COMMOT, CellNEST, and Giotto in all five sections, while remaining similar to CellChat v2 (S4 Fig; S12 Table). Because the same scRNA-seq data also contributed to SpaHCC construction, we interpret this analysis as an internal directional-resolution assessment rather than an independent validation of communication accuracy.

### Cross-patient stability, receptor-state coupling, and representative external spatial case studies

To evaluate the consistency of the model results across patients and their relationship with changes in cellular states, we integrated and analyzed the patient-level enrichment results from the Alzheimer’s disease (AD) group. The results showed that significant signals were primarily concentrated in inhibitory neurons and excitatory neurons, while a smaller number of significant entries were also detected in astrocytes (Fig 4A). Representative ligand–receptor combinations included CADM1–CADM1, CNTN1–NRCAM, NCAM1–NCAM1, JAM3–JAM3, and FLRT2–ADGRL3, and the corresponding frequently enriched transcription factors included TAL1, GATA2, SOX2, GATA3, and FOXA1. Most of these molecules are related to cell adhesion, neuronal connection formation, synaptic stability, and the regulation of plasticity. Among them, NCAM-, CADM-, and contactin/NRCAM-related molecules are mainly involved in the stabilization of neuronal connectivity and synapse organization, whereas the FLRT–ADGRL complex is closely associated with cell adhesion and neural guidance [31,32]. Together with the patient-level heatmap, these results indicate that the observed signals were not driven by any single patient, but instead recurred across multiple AD patients (Fig 4B). Random-baseline analysis further supported this observation in most held-out patients (S6 Table). Specifically, five of the six held-out AD patients showed observed overlaps greater than random expectation, with enrichment ratios ranging from 1.201 to 1.582 and empirical *P*-values of 0.00050, whereas Patient 53 showed an overlap close to random expectation. These results suggest that the key signals identified by the model exhibited cross-patient stability in most patients rather than being mainly explained by random overlap.

**Fig 4:**
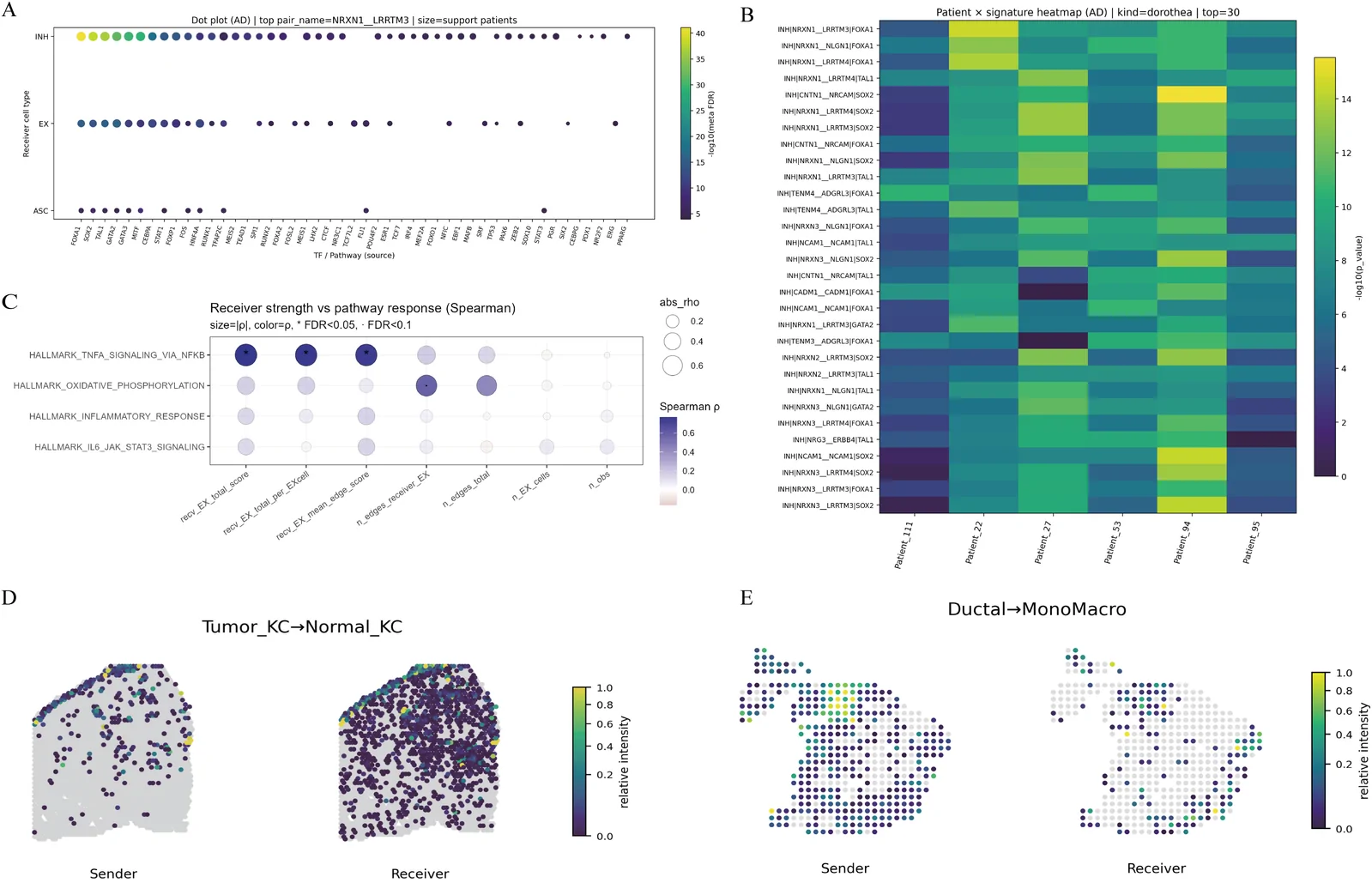
Cross-patient stability, receiver-state coupling, and representative spatial cases from external disease datasets. **(A)** Patient-level ranking of candidate enriched signals in the AD cohort. The x-axis shows the overall ranking score, and the y-axis lists the corresponding cell type, ligand-receptor pair, and transcription factor combinations. **(B)** Heatmap showing the patient-level occurrence and relative enrichment patterns of representative candidate signals across AD patients. **(C)** Correlation analysis between receiver signaling strength and functional-state changes in excitatory neurons. Colors indicate Spearman’s correlation coefficients, and dot size indicates the magnitude of the correlation across multiple incoming-signal metrics and functional programs. **(D)** Representative spatial case in cSCC, showing sender-side and receiver-side spatial distributions of the interaction between tumor-associated keratinocytes and normal keratinocytes. **(E)** Representative spatial case in PDAC, showing sender-side and receiver-side spatial distributions of the interaction between ductal-like cells and monocyte/macrophage populations.

At the patient level, we further examined the relationship between incoming communication signals received by excitatory neurons and changes in their pathway activity. The results showed that stronger received signals were generally associated with higher activity of inflammation-related pathways (Fig 4C), including TNFA signaling via NF-κB, inflammatory response, and IL6–JAK–STAT3 signaling; oxidative phosphorylation showed a similar trend as well. Moreover, this pattern was largely consistent across different strategies for quantifying received signal intensity. Taken together, these results suggest that in AD, stronger incoming signals to excitatory neurons are associated with a more pronounced inflammation-related state, consistent with previous studies reporting persistent neuroinflammatory activation in the disease [33,34]. This analysis was used to evaluate whether SpaHCC learned communication patterns associated with receiver-side cellular states. To further assess whether these patterns were solely driven by the direct use of downstream-state features, we performed a SpaHCC-noDS ablation analysis, in which the receiver-side differential-signaling component was removed from the model; the ablation results are provided in S7 Table.

To further illustrate the spatial interpretability of the model in external disease settings, we presented one representative case from each of the cSCC and PDAC datasets. In cSCC, the sender-side signal of the interaction between tumor-associated keratinocytes and normal keratinocytes showed prominent local clustering, whereas the receiver-side signal was enhanced in neighboring regions (Fig 4D), suggesting that such interactions are more likely to occur within local microenvironments. This is consistent with previous reports showing that the cellular composition and spatial neighborhoods in cSCC exhibit localized organization [35]. In PDAC, the interaction between ductal-like cells and macrophages also displayed a clear local spatial pattern (Fig 4E), suggesting that this type of interaction is more likely to occur within the local tumor microenvironment, in agreement with the established role of macrophages as important components of the PDAC microenvironment [36]. Together, these results indicate that SpaHCC identified cross-patient-stable candidate signals in AD, characterized their associations with inflammation-related state changes, and localized representative candidate cell–cell interactions with spatially restricted patterns in external disease contexts.

## Discussion

In this study, we developed SpaHCC to address three major challenges in single-cell-resolution communication inference from spot-based spatial transcriptomics, namely uncertain cell-to-spot mapping, limited use of local spatial context, and false positives introduced by ligand-receptor co-expression alone. By integrating single-cell transcriptomics, spatial transcriptomics, and prior ligand-receptor knowledge into a unified heterogeneous graph, SpaHCC identifies and characterizes candidate communication events using molecular features, spatial neighborhood information, and cellular functional states. Within this framework, SpaHCC integrates spatial context with pathway activity, transcription factor activity, and receptor-side response information to support spatially informed prioritization and biological contextualization of candidate communication events [2,7,37]. This positioning is consistent with recent overviews of the CCC field, which emphasize that newer methods are increasingly expected to jointly model spatial context, intracellular responses, and more complex sender-receiver structures rather than relying on ligand-receptor co-expression alone [8,14,15].

Applied to Alzheimer’s disease data, SpaHCC identified candidate communication patterns centered on excitatory neuron interactions and further highlighted candidate modules related to NRXN and FLRT2, together with their potential associations with inflammation-associated transcriptional programs. These findings suggest that the candidate communication patterns inferred from the Alzheimer’s disease dataset are unlikely to be explained by changes in individual ligand–receptor pairs alone, but may instead reflect coordinated alterations in neuronal connectivity remodeling, local inflammatory activation, and cell-state transitions. Recent studies have shown that Alzheimer’s disease is accompanied by substantial transcriptional remodeling across neuronal and glial populations, as well as spatially heterogeneous changes associated with disease progression [38–40]. More recent spatial and single-nucleus studies further revealed cortical layer-restricted pathology-associated programs, cell-type-specific heterogeneity in neuronal and glial populations, and spatially localized transcriptomic alterations linked to Alzheimer’s disease pathology [41,42]. In this context, our results further suggest that increased communication involving excitatory neurons may reflect not only local spatial proximity, but also dysregulated molecular programs related to synaptic maintenance, circuit remodeling, and inflammatory responses.

Despite its performance in Alzheimer’s disease data and representative external cSCC and PDAC case studies, SpaHCC still has several limitations. First, its performance depends on the quality of cross-modal alignment between single-cell and spatial transcriptomic data, and errors in cell-to-spot mapping may propagate to downstream communication inference. For spot-based platforms such as Visium, spatial resolution and spot-level mixing may further affect the localization and interpretation of sender and receiver cells. Sensitivity analyses of relay-field parameters and cell-to-spot mapping perturbations are provided in S3 and S4 Tables, respectively, and support the partial stability of the strongest inferred LR signals under moderate parameter or mapping changes. Second, differences among patients in sequencing depth, pathological stage, cell composition, and spatial slice quality may influence comparisons of communication strength across samples. Although normalization, cell-type-level summarization, cross-patient stability analysis, and random-baseline analysis help reduce these effects, they cannot completely eliminate patient-level heterogeneity. Third, SpaHCC depends on curated ligand–receptor resources to define the candidate interaction universe. Therefore, candidate communication events involving ligand–receptor relationships absent from current databases may not be recovered. Finally, although the COMMOT simulated benchmark provides a ground-truth-based evaluation of model recovery performance, the candidate communication events inferred from real disease datasets remain computational predictions and do not by themselves establish causality. Future work could extend SpaHCC by integrating higher-resolution spatial transcriptomics, spatial multi-omics data, more comprehensive ligand–receptor resources, and perturbation or imaging-based validation strategies for communication-driven cell-state transitions, thereby enabling a more systematic analysis of spatiotemporal regulation in complex diseases [43,44].

## Materials and methods

### Mapping cells to spatial spots

We used Tangram to align single-cell transcriptomic data with spatial transcriptomic data, yielding a soft assignment matrix 𝐏 from cells to spatial spots [16]. Let $N_{c}$ denote the number of cells and $N_{s}$ denote the number of spatial spots. Then,


$$\begin{array}{c} 𝐏\in {\mathbb{R}}^{N_{c}\times N_{s}}. \end{array}$$ (1)

Here, $P_{cu}$ denotes the probability of assigning cell *c* to spatial spot *u*. Because the initial mapping weights may vary across cells, we performed row-wise normalization on the assignment matrix to eliminate this effect and ensure comparability across cells, such that the assignment probabilities for each cell sum to 1:


$$\begin{array}{c} P_{cu}\leftarrow \frac{P_{cu}}{\sum_{u^{\prime }=1}^{N_{s}}P_{cu^{\prime }}}. \end{array}$$ (2)

Because the output of Tangram is typically dense, we further pruned candidate spatial spots for each cell by retaining only those with assignment probabilities greater than a threshold η, while restricting each cell to be connected to at most *M* spatial spots. By default, we set the minimum probability threshold to 0.01 and retained up to three candidate spatial spots for each cell.

### Cell and spot node feature construction

To jointly characterize cell states and the spatial environment, we constructed node features for cells and spatial spots, respectively. For cell nodes, the input feature is formed by concatenating four components: a low-dimensional single-cell representation, a one-hot encoding of cell type, and pathway activity and transcription factor activity scores computed using decoupleR based on the PROGENy and DoRothEA networks [17–19]. Let ${𝐳}_{i}^{expr}$, ${𝐳}_{i}^{ct}$, ${𝐳}_{i}^{pw}$, and ${𝐳}_{i}^{tf}$ denote the expression PCA embedding, cell-type encoding, pathway activity, and transcription factor activity of the *i*th cell, respectively. The cell node feature is then defined as


$$\begin{array}{c} {𝐱}_{i}^{cell}=\left[{𝐳}_{i}^{expr}\;\|\;{𝐳}_{i}^{ct}\;\|\;{𝐳}_{i}^{pw}\;\|\;{𝐳}_{i}^{tf}\right]. \end{array}$$ (3)

The cell expression features were obtained by applying principal component analysis (PCA) to the gene expression matrix derived from single-cell transcriptomic data (scRNA-seq/snRNA-seq), and were then concatenated with the cell-type one-hot encoding, PROGENy scores, and DoRothEA scores to form the final cell feature matrix.

For spatial spot nodes, we first applied PCA to the spot-level gene expression matrix. We then aggregated cell-level pathway activity and transcription factor activity scores to the spot level through the Tangram mapping. Let 𝐏 denote the soft assignment matrix from cells to spatial spots, and let ${𝐙}^{cell}$ denote the matrix of cell-level functional scores. The corresponding spot-level functional score matrix is given by


$$\begin{array}{c} {𝐙}^{spot}={𝐏}^{⊤}{𝐙}^{cell}. \end{array}$$ (4)

We then concatenated the low-dimensional expression feature ${𝐳}_{j}^{expr}$, pathway activity ${𝐳}_{j}^{pw}$, and transcription factor activity ${𝐳}_{j}^{tf}$ of each spatial spot with the niche feature ${𝐳}_{j}^{niche}$ obtained by averaging over neighboring spots, the graph Laplacian positional encoding ${𝐳}_{j}^{pe}$, and the normalized two-dimensional coordinate ${𝐳}_{j}^{xy}$, to obtain the final feature representation of spot node *j*:


$$\begin{array}{c} {𝐱}_{j}^{spot}=\left[{𝐳}_{j}^{expr}\;\|\;{𝐳}_{j}^{pw}\;\|\;{𝐳}_{j}^{tf}\;\|\;{𝐳}_{j}^{niche}\;\|\;{𝐳}_{j}^{pe}\;\|\;{𝐳}_{j}^{xy}\right]. \end{array}$$ (5)

### Ligand–receptor node construction and feature representation

Each ligand–receptor (LR) node in SpaHCC represents one curated ligand–receptor interaction rather than an individual ligand or receptor gene. LR nodes were constructed from a unified LR dictionary compiled from prior ligand–receptor resources. Before feature construction, ligand and receptor subunit fields were standardized by parsing multiple possible delimiters, removing empty or duplicated entries, and generating consistent ligand-subunit and receptor-subunit annotations. For multimeric ligands or receptors, the full subunit composition was retained as node metadata.

The LR node features included both structural and functional annotations. Structural features included whether the interaction involved a multimeric complex, the number of ligand subunits, the number of receptor subunits, and the log-transformed total number of subunits. Functional annotations were encoded as categorical features, including ligand location, receptor location, receptor class, and interaction mode. Ligand and receptor locations were grouped into secreted, membrane, extracellular matrix, and other categories, whereas interaction modes were grouped into paracrine, juxtacrine, extracellular-matrix-mediated, and other categories.

The LR node set was defined by the standardized curated LR dictionary and was not dynamically determined by dataset-specific expression patterns. Expression information was instead incorporated in downstream cell–LR edge construction, spot–LR spatial support calculation, and sender cell–LR–receiver cell triad scoring. This design keeps LR node identities consistent across datasets while allowing dataset-specific ligand and receptor expression patterns to affect communication inference.

### Multi-relational edge construction

After constructing the node features, we established multiple types of relational edges among cells, spatial spots, and ligand–receptor (LR) pairs. First, based on the expression of ligand or receptor subunits in single cells, we constructed sender-side and receiver-side relational edges, respectively. For the ℓth LR pair, let ${ℒ}_{\ell }$ and ${ℛ}_{\ell }$ denote the sets of ligand subunits and receptor subunits, respectively. The aggregated expression of cell *i* on the sender side and receiver side is defined as


$$\begin{array}{c} s_{i\ell }^{\left(L\right)}=Agg\{x_{ig}:g\in {ℒ}_{\ell }\},\;s_{i\ell }^{\left(R\right)}=Agg\{x_{ig}:g\in {ℛ}_{\ell }\}. \end{array}$$ (6)

Here, Agg denotes the minimum operator across subunit expression values. These relational edges were retained only when the corresponding LR pair satisfied the cell-type detection-rate criterion and the single-cell expression threshold. CellChat and CellPhoneDB were not jointly required to support the same candidate relation in this prior-filtering step. For the AD analysis reported here, CellChat was applied to the scRNA-seq data to generate the prior filtering tables, with significant interactions defined at P≤0.05. Each significant interaction was separated into an (LR pair,source cell type) sender-side prior and an (LR pair,target cell type) receiver-side prior. Because the sender-side and receiver-side priors were matched independently, the exact source–LR–target triplet was not required to occur as a single CellChat record. Candidate cell→LR and LR→cell edges were then filtered separately according to the corresponding sender-side and receiver-side prior tables [28,45].

In addition to the relations between cells and LR nodes, we also constructed support edges between spatial spots and LR nodes, as well as neighborhood edges among spatial spots. For the ℓth LR pair, let ${ℒ}_{\ell }$ denote the set of ligand subunits. SpaHCC first computes a ligand-side raw support signal at each spatial spot *u*:


$$\begin{array}{c} h_{\ell }(u)=Agg\{x_{ug}:g\in {ℒ}_{\ell }\}. \end{array}$$ (7)

Here, $x_{ug}$ denotes the normalized expression of ligand gene *g* in spot *u*, and A*gg* denotes the subunit aggregation function. For multimeric ligands, the minimum operator was used by default.

To mitigate local detection dropout and random noise, we transformed the raw support vector into a relay field by iterative spatial smoothing over the spot-neighborhood graph:


$$\begin{array}{c} {𝐫}_{\ell }^{\left(0\right)}={𝐡}_{\ell },\;{𝐫}_{\ell }^{\left(t+1\right)}=(1-\alpha ){𝐫}_{\ell }^{\left(t\right)}+\alpha \tilde{𝐀}{𝐫}_{\ell }^{\left(t\right)},\;t=0,...,K-1. \end{array}$$ (8)

Here, ${𝐡}_{\ell }=[h_{\ell }(1),...,h_{\ell }(N_{s}){]}^{⊤}$ denotes the raw ligand-side support vector of the ℓth LR pair across all spatial spots, and $\tilde{𝐀}={𝐃}^{-1}𝐖$ is the row-normalized spot adjacency matrix. In the default setting, the spot-neighborhood graph was constructed as an undirected k-nearest-neighbor graph using spatial coordinates, with *k* = 6. The smoothing coefficient was set to α=0.6, and the number of relay iterations was set to *K* = 2. The final relay field is denoted as ${𝐫}_{\ell }={𝐫}_{\ell }^{\left(K\right)}$.

Based on ${𝐫}_{\ell }$, we constructed support edges from spatial spots to LR nodes and generated reverse LR-to-spot edges. To avoid overly dense spot–LR connections, we retained at most the top 200 supporting spots for each LR pair and at most the top 50 LR pairs for each spot. The edge weight between spot *u* and LR pair ℓ was defined as


$$\begin{array}{c} w(u,\ell )=r_{\ell }(u). \end{array}$$ (9)

The spot–LR support edge was defined as a ligand-side spatial support term. Receptor-side evidence was not directly used to construct this edge, but was modeled separately through direction-aware LR-to-cell receiver edges and integrated during sender cell–LR–receiver cell triad scoring. In addition, higher-order spot structural relations were derived from shortest-path distances on the spot graph, yielding *spdist1*, *spdist2*, and *spdist3* relations for the heterogeneous graph encoder.

### Heterogeneous graph representation learning and model training

We used a heterogeneous graph encoder to learn representations for the three types of nodes: cells, spatial spots, and ligand–receptor (LR) pairs. This encoding process follows the modeling paradigm of the heterogeneous graph Transformer, in which message passing is performed separately for each relation type and the resulting multi-relational information is subsequently integrated [20]. The node update rule of the heterogeneous graph neural network is defined as


$$\begin{array}{c} {𝐡}_{v}^{\left(l+1\right)}=HeteroConv\left(\left\{{TransformerConv}_{r}\left({𝐡}_{u}^{\left(l\right)},e_{uv}\right):r\in ℛ\right\}\right). \end{array}$$ (10)

Here, ${𝐡}_{v}^{\left(l\right)}$ denotes the representation of node *v* at the *l*th layer, ℛ denotes the set of re*l*ation types, and $e_{uv}$ represents the edge attributes. In this process, attention-based message passing is first performed separately for each relation type, after which information from different relations is aggregated to produce node embeddings that jointly capture molecular relationships, spatial structure, and mapping information.

Model training involved two stages: self-supervised contrastive pretraining and link-prediction-based fine-tuning. Specifically, we performed two stochastic forward passes on the same sampled heterogeneous mini-batch to obtain two sets of node embeddings, ${𝐳}^{\left(1\right)}$ and ${𝐳}^{\left(2\right)}$, and used the InfoNCE loss to enforce consistency between the representations of the same node across the two passes:


$$\begin{array}{c} {ℒ}_{pre}=\frac{1}{\left|𝒯\right|}\sum_{t\in 𝒯}CE\left(\frac{norm({𝐳}_{t}^{\left(1\right)})norm({𝐳}_{t}^{\left(2\right)}{)}^{⊤}}{\tau },𝐈\right). \end{array}$$ (11)

Here, 𝒯 denotes the set of node types, and τ is the temperature parameter. Because instance-level contrastive learning may include biologically similar cells as negative samples, we further evaluated a false-negative-aware masked InfoNCE variant across 12 patient samples. Specifically, among the sampled negative cells for each anchor cell, cells with the same annotated cell type as the anchor were treated as plausible false negatives and excluded from the InfoNCE negative set, whereas negative cells from other cell types were retained. The positive-pair definition, model architecture, data inputs, and other training settings were kept unchanged between the original and masked formulations. Embedding-level properties and downstream LR-ranking robustness were then compared between the two formulations; the results are provided in S9 Table.

After pretraining, the pretrained encoder parameters were used to initialize the fine-tuning stage. During fine-tuning, the model was optimized using a link-prediction objective on the target relations. Observed graph relations constructed from prior-supported candidate evidence were treated as pseudo-positive edge labels, while negative edges were generated online by negative sampling from unobserved relations. These pseudo-positive labels served as weak-supervision signals for representation refinement. The contribution of the BCE edge-label fine-tuning step was further assessed using a no-finetuning ablation while keeping the same heterogeneous graph construction; the results are provided in S10 Table. For each sampled edge (*u*,*v*), the predicted probability is defined as


$$\begin{array}{c} p_{uv}=\sigma ({𝐡}_{u}^{⊤}{𝐡}_{v}). \end{array}$$ (12)

Because pseudo-positive edge labels were relatively sparse among the sampled training edges, we optimized the model during fine-tuning using a binary cross-entropy loss with class-imbalance correction:


$$\begin{array}{c} {ℒ}_{link}=-\sum_{\left(u,v\right)}\left[\omega y_{uv}\log p_{uv}+(1-y_{uv})\log(1-p_{uv})\right], \end{array}$$ (13)

where $y_{uv}\in \{0,1\}$ denotes the pseudo-positive or sampled-negative edge label, $p_{uv}$ denotes the predicted probability of the model, and $\omega =N_{neg}/N_{pos}$ is the positive-class weight computed from the numbers of sampled negative and pseudo-positive edges in each training batch.

In addition, to better constrain the node embeddings to preserve functional-state information, we introduced auxiliary prediction losses for pathway activity and transcription factor activity for both cells and spatial spots, and optimized them using mean squared error. During fine-tuning, the overall optimization objective is defined as


$$\begin{array}{c} {ℒ}_{ft}={ℒ}_{link}+{ℒ}_{aux}, \end{array}$$ (14)

$$\begin{array}{c} {ℒ}_{aux}=\lambda_{aux}\cdot \frac{\lambda_{path}{ℒ}_{path}+\lambda_{tf}{ℒ}_{tf}}{T}. \end{array}$$ (15)

Here, ${ℒ}_{path}$ and ${ℒ}_{tf}$ denote the reconstruction losses for pathway activity and transcription factor activity, respectively, and *T* denotes the number of auxiliary objectives that are actually enabled. The coefficients $\lambda_{aux}$, $\lambda_{path}$, and $\lambda_{tf}$ control the weights of the overall auxiliary loss, the pathway loss, and the transcription factor loss, respectively. A pretraining-then-fine-tuning strategy has been shown to improve the representation quality of graph neural networks and their downstream generalization performance [21].

### Spatial relay-based triad scoring and iterative screening

After learning embeddings for cells, spatial spots, and ligand–receptor (LR) pairs, we scored candidate communication events at the level of sender cell–LR pair–receiver cell triads. For a sender cell $c_{s}$, a receiver cell $c_{t}$, and the ℓth LR pair, we first computed the basic matching scores on the sender side and the receiver side as follows:


$$\begin{array}{c} {base}_{s}(c_{s},\ell )=\sigma \left(\frac{{𝐞}_{c_{s}}^{cell}\cdot {𝐞}_{\ell }^{LR}}{\tau_{d}}\right),\;{base}_{t}(\ell ,c_{t})=\sigma \left(\frac{{𝐞}_{\ell }^{LR}\cdot {𝐞}_{c_{t}}^{cell}}{\tau_{d}}\right). \end{array}$$ (16)

Here, σ(·) denotes the sigmoid function, and $\tau_{d}$ is the temperature parameter.

We then computed the element-wise product between the probability of mapping a cell to each spot and the relay field of the corresponding LR pair over those spots, and took the maximum value as the relay score:


$$\begin{array}{c} {Relay}_{s}(c_{s},\ell )=\max_{u\in 𝒮(c_{s})}P_{c_{s}u}R_{\ell }(u), \end{array}$$ (17)

$$\begin{array}{c} {Relay}_{t}(\ell ,c_{t})=\max_{v\in 𝒮(c_{t})}P_{c_{t}v}R_{\ell }(v). \end{array}$$ (18)

Here, $P_{cu}$ denotes the probability of assigning cell *c* to spot *u*, and $R_{\ell }(u)$ denotes the relay field intensity of the ℓth LR pair at spot *u*, defined as


$$\begin{array}{c} R_{\ell }(u)=[{𝐫}_{\ell }{]}_{u}. \end{array}$$ (19)

Here, ${𝐫}_{\ell }$ denotes the final relay field of the ℓth LR pair after *K* steps of spatial smoothing as defined in the relay-field smoothing step above. The final triad score is defined as


$$\begin{array}{c} S(c_{s},\ell ,c_{t})={base}_{s}(c_{s},\ell )\cdot {Relay}_{s}(c_{s},\ell )\cdot {base}_{t}(\ell ,c_{t})\cdot {Relay}_{t}(\ell ,c_{t})\cdot Bridge(c_{s},c_{t}). \end{array}$$ (20)

Here, $Bridge(c_{s},c_{t})$ is an optional spatial bridging term that characterizes the spatial relationship between the sender cell and the receiver cell. When this term is not enabled, spatial information is still incorporated into the triad score through the sender-side and receiver-side relay scores. When it is enabled, the model further constrains the spatial compatibility between the sender and receiver cells, thereby more stringently reducing spatially implausible false-positive candidates. For datasets in which the communication labels are not defined by local spatial-neighborhood enrichment, SpaHCC can disable the direct spatial-neighborhood terms in the final triad score. In this setting, the sender-side relay term, receiver-side relay term, and optional sender–receiver bridging term are set to one:


$$\begin{array}{c} {Relay}_{s}(c_{s},\ell )=1,\;{Relay}_{t}(\ell ,c_{t})=1,\;Bridge(c_{s},c_{t})=1. \end{array}$$ (21)

The resulting score is therefore


$$\begin{array}{c} S_{nonspatial}(c_{s},\ell ,c_{t})={base}_{s}(c_{s},\ell )\cdot {base}_{t}(\ell ,c_{t}). \end{array}$$ (22)

This setting retains the LR-mediated sender–receiver structure of the original SpaHCC triad score, while excluding the direct contribution of local spatial-neighborhood terms from the final score. In this study, this scoring setting was used for the COMMOT simulated data, which provide labeled sender–receiver–LR communication events. For each LR pair, we retained high-scoring sender cell–receiver cell combinations to form candidate triads, and ranked LR pairs according to either the maximum score or the average score of these high-scoring triads. The top-ranked LR pairs were then fed back into the next round of edge filtering, model retraining, and re-scoring, thereby forming an iterative graph-refinement procedure. The contribution of this iterative refinement was further assessed by comparing single-round and multi-round inference results (S5 Table).

### Ground-truth benchmark and spatial consistency evaluation

To assess whether the inferred results are consistent with expected spatial communication patterns, we used the distance enrichment score (DES) as a metric of spatial consistency [7,27]. For each candidate ligand–receptor pair ℓ, we first computed the observed Wasserstein distance based on the spatial expression distributions of the ligand side and the receptor side, denoted by $d_{real}(\ell )$. We then constructed a random background by randomly permuting the spatial coordinates of the ligand and receptor sides, and repeatedly recalculated the Wasserstein distance to obtain a set of simulated distances,


$$\begin{array}{c} \{d_{sim}^{\left(1\right)}(\ell ),...,d_{sim}^{\left(n\right)}(\ell )\}. \end{array}$$

Based on these quantities, the spatial communication tendency of the ligand–receptor pair was defined as


$$\begin{array}{c} d_{ratio}(\ell )=\frac{d_{real}(\ell )}{\frac{1}{n}\sum_{b=1}^{n}d_{sim}^{\left(b\right)}(\ell )}. \end{array}$$ (23)

When $d_{ratio}(\ell )$ is small, the sender side and receiver side of the corresponding ligand–receptor pair are spatially closer, indicating a greater tendency toward short-range communication; conversely, a larger value suggests a tendency toward long-range communication. We then constructed a null distribution based on the simulated distance set and used a one-sided permutation test to assess significance, thereby distinguishing short-range from long-range spatial communication tendencies.

After obtaining the spatial communication tendency and significance for all ligand–receptor pairs, we constructed an expected short-range communication list $L_{s}$ and an expected long-range communication list $L_{f}$, respectively, and extracted the observed communication list *S* from the predictions of different methods. We then evaluated the consistency between the expected spatial tendency and the observed communication results by progressively accumulating the weighted proportions of matched and unmatched interactions along the expected communication list. For short-range communication, let $P_{match}(S_{n},j)$ and $P_{unmatch}(S_{n},j)$ denote the cumulative proportions of matched and unmatched interactions, respectively, among the top *j* interactions. The DES is then defined as


$$\begin{array}{c} DES=\max_{j}\left[P_{match}(S_{n},j)-P_{unmatch}(S_{n},j)\right]. \end{array}$$ (24)

A higher DES indicates better agreement between the communication patterns predicted by a given method and the expected spatial distance trend.

### Receiver-side transcriptional response analysis for model interpretation

To evaluate whether high-scoring candidate communication events are associated with molecular state changes on the receiver side, we analyzed the downstream transcriptional responses of receiver cells. For a given receiver cell $c_{i}$ and candidate ligand–receptor pair ℓ, the received signal intensity is defined as the sum of the scores of all high-confidence triads associated with ℓ that point to this cell:


$$\begin{array}{c} R_{i\ell }=\sum_{(c_{s},\ell ,c_{i})\in {𝒯}_{i,\ell }}S(c_{s},\ell ,c_{i}). \end{array}$$ (25)

Within each fixed receiver cell type, cells were grouped according to their received signal intensity, and over-representation analysis (ORA) was performed on the differentially expressed genes in the high-signal group relative to the low-signal group, in order to identify receptor-side pathway and transcription factor responses associated with the candidate communication events. Pathway and transcription factor annotations were obtained from PROGENy and DoRothEA, respectively [18,19].

To assess cross-patient stability, we summarized the significance results of the same receiver cell type–ligand–receptor pair–feature signature combination across different patients and combined the corresponding *P*-values using Fisher’s method:


$$\begin{array}{c} X=-2\sum_{m=1}^{M}\log p_{m}. \end{array}$$ (26)

Here, *M* is the number of patients included in the integration, and $p_{m}$ denotes the significance level in the *m*th patient. To further evaluate the cross-patient stability of the results, we performed leave-one-patient-out (LOPO) validation and constructed a random baseline. Let $S_{train}$ denote the set of consensus results from the training set, and let $S_{test}$ denote the set of significant results in the held-out patient. The observed overlap between the two sets is defined as


$$\begin{array}{c} O=\left|S_{train}\cap S_{test}\right|. \end{array}$$ (27)

Let *A* denote the consensus result set from the training set, *B* denote the set of significant results in the held-out patient, and *U* denote the size of the full background universe. The expected overlap under the random background is defined as


$$\begin{array}{c} E_{random}=\frac{\left|A||B\right|}{\left|U\right|}. \end{array}$$ (28)

The overlap enrichment ratio is then defined as


$$\begin{array}{c} enrichment\;ratio=\frac{O}{E_{random}}. \end{array}$$ (29)

In addition, empirical significance was assessed using a permutation test. Let *O*^(*b*)^ denote the random overlap obtained in the *b*th permutation, and let $B_{perm}$ denote the total number of permutations. The empirical *P*-value is then defined as


$$\begin{array}{c} p_{emp}=\frac{1+\sum_{b=1}^{B_{perm}}1\left(O^{\left(b\right)}\geq O\right)}{B_{perm}+1}. \end{array}$$ (30)

When the observed overlap is greater than the random expectation and the empirical *P*-value is small, it suggests that the key results repeatedly detected across patients are unlikely to be explained primarily by random overlap.

### Coupling analysis between patient-level received signal intensity and pathway responses

We analyzed the relationship, at the patient level, between the strength of communication signals received by excitatory neurons and the associated changes in pathway activity. For the *d*th patient, let $X_{d}$ denote the received signal intensity of excitatory neurons, and let $Y_{d,\gamma }$ denote the score of pathway γ. The patient-level correlation for pathway γ is defined as


$$\begin{array}{c} \rho_{\gamma }={corr}_{Spearman}\left(\{X_{d}{\}}_{d=1}^{D},\{Y_{d,\gamma }{\}}_{d=1}^{D}\right). \end{array}$$ (31)

We calculated the Spearman correlation between received signal intensity and each candidate pathway separately, and adjusted the resulting *P*-values for multiple testing using the Benjamini–Hochberg method.

## Supporting information

S1 Fig Spatial visualization of representative ligand–receptor pairs in the AD cohort. Sender-side and receiver-side spatial signal distributions inferred by SpaHCC are shown for representative ligand–receptor pairs in the AD cohort. These examples provide support that SpaHCC identifies spatially localized and pair-specific communication patterns across multiple candidate interactions.
(DOCX)

S2 Fig SpaHCC–Giotto spatial comparison and Giotto spatial-parameter sensitivity analysis. (A) Spatial visualization of the representative ligand–receptor pair NRXN1–CLSTN1 in an AD sample, comparing SpaHCC and Giotto. SpaHCC identified more localized high-scoring regions on both the sender and receiver sides, whereas Giotto showed broader and smoother spatial patterns. (B) Overall Giotto spatial metrics across alternative spatial-parameter settings, including the number of inferred interactions, mean spatial ligand–receptor expression score, number of spatially significant interactions at *P* < 0.05, and the fraction of spatially significant interactions. (C) Giotto spatial-parameter sensitivity for NRXN1–CLSTN1 across the original, strict, medium, and loose Giotto parameter settings.
(DOCX)

S3 Fig Multi-method concordance and exact directional event reprioritization. (A) Median Spearman correlations between SpaHCC and four spatial CCC comparators under the pairwise shared and all-five strict event universes. (B) SpaHCC percentile minus the median percentile of the four comparator methods for exact directional candidate events derived from the original SpaHCC biological analysis across evaluable sections.
(DOCX)

S4 FigDirectional resolution, receiver-state context, and CellChat prior-source sensitivity.(A) Conservative sender–receiver support under a fixed LR identity. (B) Exploratory receiver-state context for EX→EFNA5–EPHA4→EX. (C) Section-specific relationship between the scRNA-seq-derived CellChat prior-source ordering and final SpaHCC ranking.(DOCX)

S1 Table Key parameter settings used in SpaHCC. This table summarizes the key parameter settings used in the main SpaHCC analyses, including cell-to-spot mapping thresholds, spatial relay parameters, graph construction settings, model training settings, and iterative refinement settings.
(DOCX)

S2 Table Software versions and settings used for comparison methods. This table summarizes the software versions, major parameter settings, and analysis modes used for the compared cell–cell communication methods.
(DOCX)

S3 Table Relay-field parameter sensitivity analysis. This table reports the sensitivity analysis of relay-field parameters, including the smoothing coefficient, number of smoothing steps, and spot-neighborhood graph size.
(DOCX)

S4 Table Cell-to-spot mapping perturbation sensitivity analysis. This table reports the sensitivity of SpaHCC ligand–receptor rankings to perturbations of the Tangram-derived cell-to-spot mapping probabilities.
(DOCX)

S5 Table Single-round versus multi-round SpaHCC inference analysis. This table compares single-round and multi-round SpaHCC inference results to assess the contribution of iterative graph refinement.
(DOCX)

S6 Table Random-baseline comparison of leave-one-patient-out validation in AD. This table compares the observed leave-one-patient-out overlap with a random baseline across AD patients. The signature universe size was fixed at *U* = 16886 for all AD patients, and empirical *P*-values were calculated using 2000 permutations.
(DOCX)

S7 Table SpaHCC-noDS ablation analysis of LOPO-validated regulatory programs and ligand–receptor pairs in AD. This table summarizes the ablation analysis comparing the full SpaHCC model with SpaHCC-noDS, in which the receiver-side differential-signaling component was removed. (DOCX)

S8 TableMulti-method matched sender–LR–receiver comparison in AD sections.This table reports the harmonized five-method event universes, concordance summaries, recurrence of candidate events derived from the original SpaHCC biological analysis, and complete five-method scores, exact ranks, and within-section percentiles for EX→NRXN3–NLGN1→EX.(DOCX)

S9 Table False-negative sensitivity analysis of SpaHCC embeddings and downstream ligand–receptor prioritization This table summarizes the comparison between the original and false-negative-aware SpaHCC models across embedding-level and downstream LR-ranking analyses. (DOCX)

S10 Table No-finetuning ablation analysis of SpaHCC ligand–receptor prioritization. This table summarizes the ablation analysis in which the BCE edge-label fine-tuning step was removed while keeping the same heterogeneous graph construction.
(DOCX)

S11 Table Sample-level AUROC values for the SpaHCC spatial ablation. Sample-level AUROC values for the newly added pure non-spatial SpaHCC and full SpaHCC variants across the simulated benchmark datasets.
(DOCX)

S12 TableDirectional resolution, cross-patient recurrence, receiver-state context, and CellChat provenance.This table summarizes fixed-LR conservative directional support, cross-patient recurrence of the EX→EX NRXN–NLGN module, exploratory receiver-state context for EX→EFNA5–EPHA4→EX, and the relationship between the scRNA-seq-derived CellChat prior source and final SpaHCC ranking.(DOCX)
