# Supplementary material for: Spatial cell-cell communication inference based on cell-spot-ligand-receptor heterogeneous graphs
Source: PLoS Comput Biol. 2026 Sep 28;22(9):e1014835. doi: 10.1371/journal.pcbi.1014835 (PMC13674867; doi:10.1371/journal.pcbi.1014835)
Supplement: Supplementary file 1 [file pcbi.1014835.s001.docx]

**S1. spatial visualization of representative ligand–receptor pairs**

**Figure S1. Spatial visualization of representative ligand–receptor pairs in the AD cohort.**

Sender-side and receiver-side spatial signal distributions inferred by SpaHCC are shown for representative ligand–receptor pairs in the AD cohort. These examples provide support that SpaHCC identifies spatially localized and pair-specific communication patterns across multiple candidate interactions. These results suggest that the inferred spatial communication remodeling is reproducible across related ligand–receptor pairs rather than being driven by a single selected interaction.
