# Supplementary material for: Spatial cell-cell communication inference based on cell-spot-ligand-receptor heterogeneous graphs
Source: PLoS Comput Biol. 2026 Sep 28;22(9):e1014835. doi: 10.1371/journal.pcbi.1014835 (PMC13674867; doi:10.1371/journal.pcbi.1014835)
Supplement: Supplementary file 2 [file pcbi.1014835.s002.docx]

**S2 Fig. SpaHCC-Giotto spatial comparison and Giotto spatial-parameter sensitivity analysis.**

S2 Fig. SpaHCC-Giotto spatial comparison and Giotto spatial-parameter sensitivity analysis. (A) Spatial visualization of the representative ligand-receptor pair NRXN1-CLSTN1 in an AD sample (Patient_95), comparing SpaHCC and Giotto. SpaHCC identified more localized high-scoring regions on both the sender and receiver sides, whereas Giotto showed broader and smoother spatial patterns. (B) Overall Giotto spatial metrics across alternative spatial-parameter settings, including the number of inferred interactions, mean spatial ligand-receptor expression score, number of spatially significant interactions at P < 0.05, and the fraction of spatially significant interactions. (C) Giotto spatial-parameter sensitivity for NRXN1-CLSTN1. The LR expression-based spatial score, spatial log2 fold change, LR spatial rank, and spatial interaction rank are shown across the original, strict, medium, and loose Giotto parameter settings. Overall interaction counts and the number of spatially significant interactions remained largely unchanged across settings, whereas selected score and rank metrics showed modest parameter-dependent variation. These results indicate that Giotto spatial parameters can affect quantitative spatial scores, but the representative SpaHCC-Giotto spatial-pattern difference is not explained solely by one specific Giotto parameter setting.
