# Supplementary material for: Spatial cell-cell communication inference based on cell-spot-ligand-receptor heterogeneous graphs
Source: PLoS Comput Biol. 2026 Sep 28;22(9):e1014835. doi: 10.1371/journal.pcbi.1014835 (PMC13674867; doi:10.1371/journal.pcbi.1014835)
Supplement: Supplementary file 3 [file pcbi.1014835.s003.docx]

**S3 Fig. Multi-method concordance and exact directional event reprioritization.**

**A**

**B**

Figure S3. Multi-method concordance and exact directional event reprioritization. (A) Median Spearman correlations between SpaHCC and COMMOT, CellNEST, Giotto, and the independently run ST-derived, cell-type-projected CellChat v2 comparator. Pairwise shared universes retain the largest exact event overlap for each method pair; the all-five strict sensitivity uses 5,637 exact sender–LR–receiver events. Method-specific event-score ordering and communication-mass ordering are shown separately. (B) For each prespecified exact event and evaluable section, the SpaHCC percentile minus the median percentile of the four comparator methods. Positive values indicate higher SpaHCC prioritization for the same exact directional event. Concordance is descriptive and low concordance alone is not interpreted as added biological value.
