# Supplementary material for: Spatial cell-cell communication inference based on cell-spot-ligand-receptor heterogeneous graphs
Source: PLoS Comput Biol. 2026 Sep 28;22(9):e1014835. doi: 10.1371/journal.pcbi.1014835 (PMC13674867; doi:10.1371/journal.pcbi.1014835)
Supplement: Supplementary file 4 [file pcbi.1014835.s004.docx]

**S4 Fig. Directional resolution, receiver-state context, and CellChat prior-source sensitivity.**

**A**

**B**

**C**

Figure S4. Directional resolution, receiver-state context, and CellChat prior-source sensitivity. (A) Conservative scRNA-seq support percentile of the top sender–receiver realization selected by each method while LR identity is held fixed. The conservative statistic is the minimum of ligand-sender AUC and receptor-receiver AUC; this is internal directional-resolution support rather than independent accuracy validation. (B) Exploratory correlation between received EX→EFNA5–EPHA4→EX signal and JAK–STAT activity in excitatory-neuron receivers, with within-context percentiles relative to other EX→EX events. This analysis is non-causal. (C) Section-specific relationship between the scRNA-seq-derived CellChat prior-source ordering and final SpaHCC ranking, together with the fraction of events shifting by at least 0.20 percentile. The prior-source analysis is distinct from the independently run ST-derived, cell-type-projected CellChat v2 comparator.
