# Supplementary material for: Spatial cell-cell communication inference based on cell-spot-ligand-receptor heterogeneous graphs
Source: PLoS Comput Biol. 2026 Sep 28;22(9):e1014835. doi: 10.1371/journal.pcbi.1014835 (PMC13674867; doi:10.1371/journal.pcbi.1014835)
Supplement: Supplementary file 5 [file pcbi.1014835.s005.docx]

**S1 Table. Key parameter settings used in SpaHCC.**

The table summarizes the key parameter settings used for the main SpaHCC analysis and related model-training steps.

| **Workflow step** | **Parameter** | **Value used** | **Description** |
| --- | --- | --- | --- |
| Tangram mapping pruning | Maximum spots retained per cell in initial mapping pruning | 50 | Maximum number of candidate spots retained for each cell before cumulative-mass filtering. |
| Tangram mapping pruning | Cumulative mapping mass | 0.85 | Retained top mapping probabilities until the cumulative probability mass reached 0.85. |
| Tangram mapping pruning | Minimum mapping probability | 0.01 | Cell--spot mapping entries below this probability were removed after row normalization. |
| Tangram mapping pruning | Pruning severity | mild | Mild filtering was used to remove low-confidence cell--spot mappings while retaining mapping uncertainty. |
| Cell-to-spot edge construction | Minimum cell-to-spot probability | 0.01 | Minimum Tangram-derived probability required for constructing cell--spot edges. |
| Cell-to-spot edge construction | Maximum spots per cell | 3 | Each cell was connected to at most three candidate spatial spots. |
| Cell node feature construction | Expression PCA dimension | 50 | Number of principal components used to summarize single-cell gene-expression features. |
| Cell node feature construction | Cell-type annotation key | cell_type | Cell-type labels were encoded as one-hot features. |
| Cell node feature construction | Downstream-state features | PROGENy and DoRothEA | Pathway and transcription-factor activity features were included in the full SpaHCC model. |
| Spot node feature construction | Spatial-neighborhood size | 6 | Spot-neighborhood graph was constructed using a local neighborhood setting with at least six neighboring spots. |
| Spot node feature construction | Positional encoding dimension | 16 | Laplacian positional encoding dimension used for spot node features. |
| Functional activity estimation | PROGENy target genes per pathway | 500 | Top 500 PROGENy target genes were used for pathway activity estimation. |
| Functional activity estimation | DoRothEA confidence levels | A/B/C | DoRothEA regulons with confidence levels A, B, and C were used for transcription-factor activity estimation. |
| Functional activity estimation | Minimum matched targets | 5 | Minimum number of matched target genes required for pathway or TF activity estimation. |
| Functional activity estimation | Random seed | 123 | Random seed used in functional activity estimation. |
| Sender-side cell--LR edge construction | Ligand detection fraction threshold | 0.20 | Minimum fraction of cells in a cell type expressing the ligand component. |
| Sender-side cell--LR edge construction | Per-cell ligand-expression cutoff | 0.20 | Minimum aggregated ligand-side expression required for sender-side cell--LR edges. |
| Sender-side cell--LR edge construction | Minimum cells per cell type | 50 | Cell types with fewer than 50 cells were excluded from sender-side edge construction. |
| Receiver-side LR--cell edge construction | Receptor detection fraction threshold | 0.20 | Minimum fraction of cells in a cell type expressing the receptor component. |
| Receiver-side LR--cell edge construction | Per-cell receptor-expression cutoff | 0.00 | Minimum aggregated receptor-side expression required for receiver-side LR--cell edges. |
| Receiver-side LR--cell edge construction | Minimum cells per cell type | 25 | Cell types with fewer than 25 cells were excluded from receiver-side edge construction. |
| CellChat prior filtering | Significance threshold | 0.05 | Significant CellChat-supported cell-type-level ligand--receptor relations were used to constrain the candidate communication space. |
| Spot--LR support edge construction | Spatial graph mode | kNN | Spot-neighborhood graph used for ligand-side spatial support propagation. |
| Spot--LR support edge construction | Number of nearest neighbors | 6 | Number of nearest neighbors used in the kNN spot-neighborhood graph. |
| Spot--LR support edge construction | Spatial smoothing coefficient | 0.6 | Smoothing coefficient used for relay-field propagation. |
| Spot--LR support edge construction | Number of relay iterations | 2 | Number of spatial relay smoothing iterations. |
| Spot--LR support edge construction | Ligand subunit aggregation | minimum | Multimeric ligand expression was aggregated using the minimum subunit expression by default. |
| Spot--LR support edge construction | Maximum spots retained per LR pair | 200 | At most the top 200 supporting spots were retained for each LR pair. |
| Spot--LR support edge construction | Maximum LR pairs retained per spot | 50 | At most the top 50 LR pairs were retained for each spot. |
| Spot--LR support edge construction | Candidate LR restriction | enabled | Spot--LR support edges were constructed for LR pairs supported on both sender and receiver sides. |
| Spot structural edge construction | Maximum shortest-path distance | 3 | Higher-order spot structural relations were constructed as spdist1, spdist2, and spdist3. |
| Spot structural edge construction | Spot graph directionality | undirected | Spot structural relations were derived from an undirected spot graph. |
| Model architecture | Hidden dimension | 128 | Hidden dimension of the heterogeneous graph encoder. |
| Model architecture | Output embedding dimension | 128 | Output node embedding dimension. |
| Model architecture | Number of attention heads | 2 | Number of attention heads in the graph transformer layers. |
| Model architecture | Dropout rate | 0.2 | Dropout used during graph model training. |
| Model training | Pretraining epochs | 5 | Number of contrastive pretraining epochs. |
| Model training | Fine-tuning epochs | 20 | Number of link-prediction fine-tuning epochs. |
| Model training | Batch size | 256 | Mini-batch size used during model training. |
| Model training | Neighbor-sampling fanout | 8 | Fanout used for graph neighbor sampling. |
| Model training | Sampling hops | 2 | Number of graph-sampling hops. |
| Model training | Mixed precision training | enabled | Automatic mixed precision was used during model training. |
| Model training | Auxiliary loss weight | 0.1 | Weight for the auxiliary objective used during graph model training. |
| Model training | Pathway auxiliary loss weight | 0.1 | Weight for the pathway auxiliary objective. |
| Model training | TF auxiliary loss weight | 0.1 | Weight for the transcription-factor auxiliary objective. |
| Model training | Training relations | secrete, bind, present, at | Edge types used for link-prediction-based fine-tuning. |
| Iterative refinement | Number of refinement rounds | 3 | SpaHCC was run for three iterative graph-refinement rounds. |
| Iterative refinement | LR retention fraction per round | 0.95 | Fraction of LR pairs retained after each refinement round. |
| Iterative refinement | LR retention metric | triad_max | LR pairs were retained according to the maximum triad score. |
| Spatial LR scoring | Relay mode | kernel | Embedding-based spatial relay was used during LR ranking. |
| Spatial LR scoring | Kernel nearest neighbors | 12 | Number of nearest neighbors used for embedding-based spatial relay. |
| Spatial LR scoring | Embedding smoothing steps | 1 | Number of smoothing steps for embedding-based spatial relay. |
| Spatial LR scoring | Cell-to-spot candidates for scoring | 10 | Maximum number of mapped spots considered per cell during spatial LR scoring. |
| Spatial LR scoring | LR-associated spots for scoring | 50 | Maximum number of LR-associated spots considered during spatial LR scoring. |
| Spatial LR scoring | Sender candidates per LR | 200 | Maximum number of sender-side candidates retained per LR during triad scoring. |
| Spatial LR scoring | Receiver candidates per LR | 200 | Maximum number of receiver-side candidates retained per LR during triad scoring. |
| Spatial LR scoring | Maximum triads per LR | 1500 | Maximum number of candidate sender--LR--receiver triads summarized per LR. |
| Spatial LR scoring | Dot-product temperature | 3.0 | Temperature used when converting embedding dot products to compatibility scores. |
