# Supplementary material for: Spatial cell-cell communication inference based on cell-spot-ligand-receptor heterogeneous graphs
Source: PLoS Comput Biol. 2026 Sep 28;22(9):e1014835. doi: 10.1371/journal.pcbi.1014835 (PMC13674867; doi:10.1371/journal.pcbi.1014835)
Supplement: Supplementary file 6 [file pcbi.1014835.s006.docx]

**S2 Table. Software versions and settings used for comparison methods.**

The table summarizes the software versions, input data, spatial settings, and main analysis settings used for comparison methods.

| **Method** | **Version used** | **Input data** | **Spatial setting** | **Main setting used in this study** |
| --- | --- | --- | --- | --- |
| CellChat | v1.6.1 | Single-cell expression matrix and cell-type annotations | Non-spatial mode | Default non-spatial CellChat workflow was used to infer significant cell-type-level ligand--receptor interactions. Significant interactions were used as prior-supported candidate relations rather than final SpaHCC communication scores. |
| CellPhoneDB | v2.1.7 | Single-cell expression matrix and cell-type annotations | Non-spatial mode | CellPhoneDB was used as a curated LR resource for LR-dictionary construction, rather than for sample-specific prior filtering in the AD analysis. |
| Giotto_expr | v1.1.2 | Spatial transcriptomics expression matrix, spatial coordinates, and cell-type annotations | Expression-based setting | Expression-based Giotto ligand--receptor scores were evaluated after mapping outputs to the shared candidate universe used in the simulated benchmark. |
| Giotto_spatial | v1.1.2 | Spatial transcriptomics expression matrix, spatial coordinates, spatial network, and cell-type annotations | Spatial mode | Giotto spatial communication analysis was performed using the spatial-network-based setting. For the Giotto spatial-parameter sensitivity analysis, the Delaunay spatial-network distance threshold was varied across original_no_cutoff, strict, medium, and loose settings. |
| COMMOT | v0.0.3 | Spatial transcriptomics expression matrix, spatial coordinates, and ligand--receptor candidate set | Spatial mode | COMMOT was run using its optimal-transport-based spatial communication framework. In the simulated benchmark, COMMOT results were mapped to the same sender--receiver--LR candidate universe and evaluated using the same ground-truth labels. |
| Scriabin | v0.0.0.9000 | Single-cell expression matrix and cell-type annotations | Original non-spatial single-cell-level setting | Scriabin was used in its original non-spatial single-cell-level configuration. No additional spatial-neighborhood constraint was introduced. |
| CellNEST | v1.0.0 | Spatial transcriptomics expression matrix, spatial coordinates, and ligand--receptor database | Spatial mode | CellNEST was run using its spatial graph-based workflow with default settings unless otherwise specified. Outputs were mapped to the shared candidate universe for quantitative comparison when applicable. |
| CytoSignal | v0.5.0 | Gene-by-spot count matrix, spatial coordinates, cluster labels, and custom ligand--receptor pairs | Spatial mode | CytoSignal was run as a manual spatial baseline on all simulated case datasets. The input matrix was formatted as genes by spots/cells. Custom ligand--receptor pairs were supplied directly, without using addIntrDB(). Spatial diffusion and contact graphs were constructed using Gaussian epsilon-based diffusion and distance-threshold contact settings. The grid step was estimated from the median nearest-neighbor distance, with eps = 3 x grid_step, sigma = grid_step, and max.r = eps. Two manual CytoSignal-style scores were generated: GauEps-Raw, using diffusion-imputed ligand expression and raw receptor expression, and GauEps-DT, using diffusion-imputed ligand expression and contact-imputed receptor expression. |
