# Supplementary material for: Spatial cell-cell communication inference based on cell-spot-ligand-receptor heterogeneous graphs
Source: PLoS Comput Biol. 2026 Sep 28;22(9):e1014835. doi: 10.1371/journal.pcbi.1014835 (PMC13674867; doi:10.1371/journal.pcbi.1014835)
Supplement: Supplementary file 7 [file pcbi.1014835.s007.docx]

**S3 Table. Sensitivity analysis of relay-field parameters in SpaHCC.**

One-parameter-at-a-time sensitivity analyses were performed using Patient_27 as a representative AD sample. The default setting was alpha = 0.6, K = 2, and k = 6. For each tested setting, LR rankings were compared with the default configuration. Pearson score correlations were not reported because the score column was unavailable for this comparison.

| **Parameter tested** | **Default setting** | **Tested setting** | **Fixed parameters** | **LR pairs retained** | **LR universe overlap with default** | **Spearman rank correlation with default** | **Top-50 overlap with default** | **Top-50 Jaccard** | **Interpretation** |
| --- | --- | --- | --- | --- | --- | --- | --- | --- | --- |
| Smoothing coefficient alpha | alpha = 0.6, K = 2, k = 6 | alpha = 0.3 | K = 2, k = 6 | 73 | 97.3% | 0.504 | 37/50 (74.0%) | 58.7% | Major top LR signals were largely preserved under weaker smoothing. |
| Smoothing coefficient alpha | alpha = 0.6, K = 2, k = 6 | alpha = 0.9 | K = 2, k = 6 | 73 | 97.3% | 0.314 | 36/50 (72.0%) | 56.3% | Major top LR signals were retained, but rank ordering was more sensitive under stronger smoothing. |
| Relay iterations K | alpha = 0.6, K = 2, k = 6 | K = 0 | alpha = 0.6, k = 6 | 73 | 95.9% | 0.174 | 36/50 (72.0%) | 56.3% | Removing spatial relay smoothing retained many top LR pairs but substantially changed their ordering. |
| Relay iterations K | alpha = 0.6, K = 2, k = 6 | K = 5 | alpha = 0.6, k = 6 | 73 | 94.5% | 0.551 | 37/50 (74.0%) | 58.7% | Extended smoothing preserved most high-ranking LR pairs, with moderate rank agreement. |
| Spot-neighborhood size k | alpha = 0.6, K = 2, k = 6 | k = 4 | alpha = 0.6, K = 2 | 73 | 97.3% | 0.647 | 41/50 (82.0%) | 69.5% | The top LR signals were most stable under a moderately smaller neighborhood size. |
| Spot-neighborhood size k | alpha = 0.6, K = 2, k = 6 | k = 8 | alpha = 0.6, K = 2 | 73 | 97.3% | 0.409 | 36/50 (72.0%) | 56.3% | Major LR signals were preserved under a larger neighborhood, although fine-scale ranking changed. |

Note: LR universe overlap was calculated as the percentage of LR pairs shared with the default setting across all ranked LR pairs. Top-50 overlap indicates the number and percentage of LR pairs shared with the top-50 LR pairs from the default setting. Top-50 Jaccard was calculated using the top-50 LR sets from the tested and default configurations.
