# Supplementary material for: Spatial cell-cell communication inference based on cell-spot-ligand-receptor heterogeneous graphs
Source: PLoS Comput Biol. 2026 Sep 28;22(9):e1014835. doi: 10.1371/journal.pcbi.1014835 (PMC13674867; doi:10.1371/journal.pcbi.1014835)
Supplement: Supplementary file 8 [file pcbi.1014835.s008.docx]

**S4 Table. Cell-to-spot mapping perturbation sensitivity analysis.**

SpaHCC ligand-receptor rankings were compared under the original Tangram mapping (P0) and 5% or 10% perturbed mapping settings (P5 and P10). The analysis focused on high-confidence LR candidates rather than reporting all individual LR indices. Across P0, P5, and P10, 69 LR pairs were shared; the top 50% cutoff corresponded to rank 37.

| **Analysis set** | **Comparison** | **n** | **Rank Spearman rho (p)** | **Score Pearson r (p)** | **Interpretation** |
| --- | --- | --- | --- | --- | --- |
| P0 top 50% | P0 vs P5 | 36 | 0.247 (p = 0.146) | 0.753 (p = 1.19 x 10^-7) | Triad scores remained correlated, but exact rank ordering was sensitive. |
| P0 top 50% | P0 vs P10 | 36 | 0.540 (p = 6.85 x 10^-4) | 0.681 (p = 4.96 x 10^-6) | Moderate rank agreement and strong score correlation were observed. |
| P0 top 50% | P5 vs P10 | 36 | 0.152 (p = 0.376) | 0.554 (p = 4.53 x 10^-4) | Scores showed correlation, whereas rank ordering remained variable. |
| Shared top 50% | P0 vs P5 | 10 | 0.891 (p = 5.42 x 10^-4) | 0.890 (p = 5.70 x 10^-4) | The LR candidates shared in the top 50% were highly consistent. |
| Shared top 50% | P0 vs P10 | 10 | 0.770 (p = 0.00922) | 0.851 (p = 0.00179) | The shared high-confidence subset showed stable ranking and scoring. |
| Shared top 50% | P5 vs P10 | 10 | 0.842 (p = 0.00222) | 0.916 (p = 1.96 x 10^-4) | The shared high-confidence subset was preserved between perturbation levels. |

Note. P0 denotes the original Tangram-derived cell-to-spot mapping. P5 and P10 denote the mappings generated after 5% and 10% perturbation of the mapping probabilities, respectively. Across P0, P5, and P10, 69 LR pairs were shared. In the P0 top-50% analysis, the top-50% cutoff was defined according to the original P0 ranking, and only P0 high-ranked LR candidates that were also present in the perturbed results were included. In the shared top-50% analysis, only LR candidates ranked within the top 50% under all three settings were included. These results support partial stability of high-confidence SpaHCC candidates, while also indicating that global LR rank ordering remains sensitive to mapping uncertainty.
