# Supplementary material for: Spatial cell-cell communication inference based on cell-spot-ligand-receptor heterogeneous graphs
Source: PLoS Comput Biol. 2026 Sep 28;22(9):e1014835. doi: 10.1371/journal.pcbi.1014835 (PMC13674867; doi:10.1371/journal.pcbi.1014835)
Supplement: Supplementary file 9 [file pcbi.1014835.s009.docx]

**S5 Table. Single-round versus multi-round SpaHCC inference analysis.**

Round 1 was treated as the single-round baseline, whereas Round 3 represented the final multi-round SpaHCC result. Pairwise comparisons were performed using LR-pair rankings based on the triad_max score in Patient 27.

| **Comparison** | **n overlapping LR pairs** | **Spearman rank rho (p)** | **Top-50 overlap** | **Top-50 Jaccard** | **Interpretation** |
| --- | --- | --- | --- | --- | --- |
| Round 1 vs Round 2 | 76 | 0.655 (p = 1.37 x 10^-10) | 37/50 (74.0%) | 58.7% | The first refinement retained many high-ranked LR pairs while changing the broader ranking. |
| Round 1 vs Round 3 | 73 | 0.488 (p = 1.21 x 10^-5) | 36/50 (72.0%) | 56.3% | The final multi-round result differed from the single-round baseline, supporting the added value of iterative graph refinement. |
| Round 2 vs Round 3 | 73 | 0.848 (p = 2.66 x 10^-21) | 45/50 (90.0%) | 81.8% | Later rounds showed stronger agreement, suggesting convergence after graph refinement. |
