# Supplementary material for: Spatial cell-cell communication inference based on cell-spot-ligand-receptor heterogeneous graphs
Source: PLoS Comput Biol. 2026 Sep 28;22(9):e1014835. doi: 10.1371/journal.pcbi.1014835 (PMC13674867; doi:10.1371/journal.pcbi.1014835)
Supplement: Supplementary file 10 [file pcbi.1014835.s010.docx]

**S6 Table. Random-baseline comparison of leave-one-patient-out validation in AD.**

Leave-one-patient-out validation was compared with a random baseline across AD patients.

| **Test patient** | **Train consensus** | **Test significant** | **Observed overlap** | **Expected overlap** | **Enrichment** | **Hypergeom. P** | **Empirical P** |
| --- | --- | --- | --- | --- | --- | --- | --- |
| Patient_27 | 3587 | 4800 | 1285 | 1019.64 | 1.260 | 7.95 x 10^-28 | 0.00050 |
| Patient_53 | 2890 | 7826 | 1337 | 1339.40 | 0.998 | 0.54719 | 0.53723 |
| Patient_95 | 4592 | 2024 | 854 | 550.41 | 1.552 | 8.91 x 10^-55 | 0.00050 |
| Patient_94 | 3250 | 6231 | 1440 | 1199.26 | 1.201 | 2.85 x 10^-22 | 0.00050 |
| Patient_22 | 4272 | 2744 | 1098 | 694.21 | 1.582 | 1.25 x 10^-77 | 0.00050 |
| Patient_111 | 4627 | 1079 | 467 | 295.66 | 1.580 | 3.40 x 10^-31 | 0.00050 |

Note. Train consensus indicates the number of consensus signatures in the training patients; Test significant indicates the number of significant signatures in the held-out patient; Observed overlap indicates the overlap between training consensus signatures and significant signatures in the held-out patient; Expected overlap indicates the expected overlap under the random baseline. The signature universe size was fixed at U = 16886 for all AD patients.
