# Supplementary material for: Spatial cell-cell communication inference based on cell-spot-ligand-receptor heterogeneous graphs
Source: PLoS Comput Biol. 2026 Sep 28;22(9):e1014835. doi: 10.1371/journal.pcbi.1014835 (PMC13674867; doi:10.1371/journal.pcbi.1014835)
Supplement: Supplementary file 11 [file pcbi.1014835.s011.docx]

**S7 Table. SpaHCC-noDS ablation analysis of LOPO-validated regulatory programs and ligand–receptor pairs in AD.**

This table summarizes the overlap between the full SpaHCC model and the SpaHCC-noDS ablation in leave-one-patient-out (LOPO) validation for the AD cohort.

**A. Overall overlap between full SpaHCC and SpaHCC-noDS**

| **Analysis level** | **Full SpaHCC count** | **SpaHCC-noDS count** | **Shared count** | **Shared / noDS** | **Interpretation** |
| --- | --- | --- | --- | --- | --- |
| TF-level LOPO validation | 43 | 13 | 10 | 76.9% | Most TFs recovered by SpaHCC-noDS were also recovered by the full model. |
| LR-pair-level LOPO validation | 35 | 27 | 19 | 70.4% | Most LR pairs recovered by SpaHCC-noDS overlapped with the full model. |

**B. Shared LOPO-validated TFs**

**TFs shared by full SpaHCC and SpaHCC-noDS**

CEBPA, EBF1, GATA2, MEIS2, MITF

RUNX1, SOX2, SPI1, STAT1, TAL1

**C. Shared LOPO-validated ligand–receptor pairs**

| **Shared LR pair** | **Shared LR pair** |
| --- | --- |
| APP__SORL1 | FLRT2__ADGRL3 |
| FLRT2__FLRT2 | NRXN1__ADGRL1 |
| NRXN1__CLSTN1 | NRXN1__LRRTM3 |
| NRXN1__LRRTM4 | NRXN2__ADGRL1 |
| NRXN2__CLSTN1 | NRXN2__LRRTM3 |
| NRXN2__LRRTM4 | NRXN3__ADGRL1 |
| NRXN3__CLSTN1 | NRXN3__LRRTM3 |
| NRXN3__LRRTM4 | TENM2__ADGRL2 |
| TENM2__ADGRL3 | TENM3__ADGRL2 |
| TENM4__ADGRL3 |  |

Note. Full SpaHCC denotes the default model, whereas SpaHCC-noDS denotes the ablated model without the receiver-side differential-signaling component. Shared / noDS was calculated as the number of TFs or LR pairs recovered by both models divided by the number recovered by SpaHCC-noDS.
