# Supplementary material for: Spatial cell-cell communication inference based on cell-spot-ligand-receptor heterogeneous graphs
Source: PLoS Comput Biol. 2026 Sep 28;22(9):e1014835. doi: 10.1371/journal.pcbi.1014835 (PMC13674867; doi:10.1371/journal.pcbi.1014835)
Supplement: Supplementary file 12 [file pcbi.1014835.s012.docx]

**S8 Table. Multi-method matched sender–LR–receiver comparison in AD sections.**

This table reports the harmonized five-method event universes, concordance summaries, prespecified candidate-event recurrence, and complete five-method details for EX→NRXN3–NLGN1→EX.

**A. All-five strict matched-event universe**

| **Section** | **Patient** | **Exact events** | **LR pairs** | **Sender–receiver pairs** |
| --- | --- | --- | --- | --- |
| S1 | Patient_27 | 1400 | 14 | 100 |
| S2 | Patient_53 | 1500 | 15 | 100 |
| S3 | Patient_95 | 1500 | 15 | 100 |
| S4 | Patient_22 | 405 | 5 | 81 |
| S5 | Patient_111 | 832 | 13 | 64 |
| Pooled | Five patients | 5637 | 16 | 100 |

**B. SpaHCC concordance with four spatial CCC comparators**

| **Comparator** | **Median pairwise shared events** | **Pairwise score ρ** | **Pairwise mass ρ** | **Strict score ρ** | **Strict mass ρ** |
| --- | --- | --- | --- | --- | --- |
| COMMOT | 2500 | 0.082 | 0.552 | 0.104 | 0.561 |
| CellNEST | 2200 | 0.112 | 0.491 | 0.150 | 0.504 |
| Giotto | 6900 | -0.043 | 0.568 | -0.085 | 0.533 |
| CellChat v2 | 6700 | 0.052 | 0.458 | 0.124 | 0.480 |

*Pairwise shared universes were used for the primary global concordance analysis; the 5,637-event all-five strict universe was used as a sensitivity analysis. For each method and section, communication mass was defined as the event-level sum of all non-negative lower-level spatial communication contributions mapped to the same sample-sender-ligand-receptor-receiver key before final method-specific score normalization. These sums were converted to within-section ranks/percentiles; absolute magnitudes were not compared across methods.*

**C. Prespecified exact directional candidate-event summary**

| **Exact event** | **Sections** | **SpaHCC median percentile** | **Comparator median percentile** | **Median difference** | **Positive sections** |
| --- | --- | --- | --- | --- | --- |
| EX → EX \| NRXN3–NLGN1 | S1;S2;S3;S5 | 0.997 | 0.269 | +0.723 | 4/4 |
| EX → EX \| EFNA5–EPHA4 | S1;S2;S3;S5 | 0.987 | 0.387 | +0.600 | 4/4 |
| EX → EX \| JAM3–JAM3 | S1;S2;S3;S5 | 0.966 | 0.470 | +0.428 | 4/4 |
| ODC → ODC \| NCAM1–FGFR1 | S1;S2;S3;S5 | 0.832 | 0.344 | +0.425 | 4/4 |

**D. Complete EX→NRXN3–NLGN1→EX five-method scores and exact ranks**

| **Section** | **Method** | **Method-specific event score** | **Exact rank/total** | **Within-section percentile** |
| --- | --- | --- | --- | --- |
| S1 | SpaHCC | 0.26455 | 2/1400 | 0.999 |
| S1 | COMMOT | 0.00596028 | 1287/1400 | 0.081 |
| S1 | CellNEST | 1.90872e-06 | 1244/1400 | 0.112 |
| S1 | Giotto | 0.0236649 | 408/1400 | 0.709 |
| S1 | CellChat v2 | 0.000128054 | 1109/1400 | 0.209 |
| S2 | SpaHCC | 0.0120489 | 21/1500 | 0.987 |
| S2 | COMMOT | 0.0120079 | 966/1500 | 0.357 |
| S2 | CellNEST | 5.998e-05 | 1224/1500 | 0.185 |
| S2 | Giotto | 0.0271625 | 376/1500 | 0.750 |
| S2 | CellChat v2 | 0.00023987 | 960/1500 | 0.361 |
| S3 | SpaHCC | 0.020214 | 5/1500 | 0.997 |
| S3 | COMMOT | 0.00729526 | 1207/1500 | 0.196 |
| S3 | CellNEST | 2.90334e-06 | 1338/1500 | 0.109 |
| S3 | Giotto | 0.0355035 | 131/1500 | 0.913 |
| S3 | CellChat v2 | 0.000286298 | 1258/1500 | 0.162 |
| S5 | SpaHCC | 0.00816401 | 3/832 | 0.998 |
| S5 | COMMOT | 0.0109209 | 476/832 | 0.429 |
| S5 | CellNEST | 5.51162e-05 | 702/832 | 0.157 |
| S5 | Giotto | 0.0289701 | 231/832 | 0.724 |
| S5 | CellChat v2 | 0.000172272 | 444/832 | 0.468 |

*The score column is reported for auditability only; absolute score magnitudes are not comparable across methods. CellChat v2 denotes the independently run ST-derived, cell-type-projected comparator.*
