# Supplementary material for: Spatial cell-cell communication inference based on cell-spot-ligand-receptor heterogeneous graphs
Source: PLoS Comput Biol. 2026 Sep 28;22(9):e1014835. doi: 10.1371/journal.pcbi.1014835 (PMC13674867; doi:10.1371/journal.pcbi.1014835)
Supplement: Supplementary file 13 [file pcbi.1014835.s013.docx]

**S9 Table. False-negative sensitivity analysis of SpaHCC embeddings and downstream ligand-receptor prioritization.**

The original InfoNCE formulation and a false-negative-aware masked formulation were compared across all 12 evaluated patients using matched cell embeddings and downstream LR-ranking outputs.

**A. Cohort-level embedding sensitivity summary**

| **Embedding metric** | **Better direction** | **Patients improved** | **Patients worsened** | **Mean delta** | **Median delta** |
| --- | --- | --- | --- | --- | --- |
| Within-/between-cell-type distance ratio | Lower | 7/12 | 5/12 | -0.0038 | -0.0051 |
| Cosine silhouette | Higher | 9/12 | 3/12 | +0.0116 | +0.0196 |
| kNN cell-type purity (k=5) | Higher | 6/12 | 6/12 | +0.0179 | +0.0017 |
| kNN cell-type purity (k=10) | Higher | 7/12 | 5/12 | +0.0188 | +0.0023 |
| kNN cell-type purity (k=20) | Higher | 7/12 | 5/12 | +0.0191 | +0.0026 |
| kNN cell-type purity (k=30) | Higher | 7/12 | 5/12 | +0.0194 | +0.0032 |
| kNN cell-type purity (k=50) | Higher | 7/12 | 5/12 | +0.0192 | +0.0038 |

**B. Patient-level changes in embedding metrics**

| **Patient** | **Condition** | **n cells** | **Delta distance ratio** | **Delta silhouette** | **Delta kNN5** | **Delta kNN10** | **Delta kNN20** | **Delta kNN30** | **Delta kNN50** |
| --- | --- | --- | --- | --- | --- | --- | --- | --- | --- |
| Patient_27 | AD | 1,382 | -0.0380 | +0.0367 | +0.0203 | +0.0225 | +0.0228 | +0.0238 | +0.0192 |
| Patient_53 | AD | 3,552 | -0.1224 | +0.0414 | +0.1026 | +0.0979 | +0.0957 | +0.0953 | +0.0944 |
| Patient_95 | AD | 3,673 | +0.0054 | +0.0009 | +0.0051 | +0.0039 | +0.0044 | +0.0047 | +0.0059 |
| Patient_12 | Control | 4,877 | +0.0832 | +0.0446 | +0.1015 | +0.0975 | +0.0951 | +0.0925 | +0.0897 |
| Patient_90 | Control | 4,686 | +0.0406 | -0.0322 | -0.0368 | -0.0404 | -0.0418 | -0.0396 | -0.0399 |
| Patient_humAD-86 | Control | 2,936 | -0.0086 | +0.0227 | +0.0331 | +0.0351 | +0.0349 | +0.0358 | +0.0351 |
| Patient_94 | AD | 5,866 | +0.0317 | -0.0674 | -0.0016 | +0.0008 | +0.0009 | +0.0018 | +0.0016 |
| Patient_22 | AD | 3,308 | +0.0416 | -0.0721 | -0.0077 | -0.0093 | -0.0097 | -0.0102 | -0.0108 |
| Patient_111 | AD | 2,309 | -0.0102 | +0.0165 | +0.0357 | +0.0418 | +0.0445 | +0.0430 | +0.0437 |
| Patient_107 | Control | 2,469 | -0.0093 | +0.0462 | -0.0067 | -0.0064 | -0.0050 | -0.0043 | -0.0026 |
| Patient_101 | Control | 4,913 | -0.0017 | +0.0107 | -0.0134 | -0.0053 | -0.0043 | -0.0046 | -0.0047 |
| Patient_28 | Control | 6,907 | -0.0584 | +0.0908 | -0.0167 | -0.0120 | -0.0087 | -0.0053 | -0.0016 |

**C. Cohort-level downstream LR-ranking robustness**

| **Metric** | **n patients** | **Mean** | **Median** | **Minimum** | **Maximum** |
| --- | --- | --- | --- | --- | --- |
| Evaluable LR-set Jaccard | 12 | 1.000 | 1.000 | 1.000 | 1.000 |
| Spearman rho, triad-mean ranking | 12 | 0.652 | 0.644 | 0.351 | 0.956 |
| Spearman rho, triad-max ranking | 12 | 0.445 | 0.486 | -0.041 | 0.846 |
| Normalized absolute rank shift, triad-mean | 12 | 0.128 | 0.122 | 0.055 | 0.290 |
| Normalized absolute rank shift, triad-max | 12 | 0.187 | 0.180 | 0.081 | 0.355 |
| Top-10 overlap, triad-mean | 12 | 45.0% | 50.0% | 0.0% | 80.0% |
| Top-10 overlap, triad-max | 12 | 28.3% | 35.0% | 0.0% | 60.0% |
| Top-20 overlap, triad-mean | 12 | 64.6% | 60.0% | 15.0% | 100.0% |
| Top-20 overlap, triad-max | 12 | 52.1% | 60.0% | 10.0% | 75.0% |
| Top-50 overlap, triad-mean | 12 | 83.5% | 82.0% | 56.0% | 100.0% |
| Top-50 overlap, triad-max | 12 | 80.0% | 86.0% | 50.0% | 100.0% |

**D. Patient-level LR rank concordance and rank shifts**

| **Patient** | **Condition** | **Common LR** | **Set Jaccard** | **rho triad-mean** | **rho triad-max** | **Median \|rank shift\| triad-mean** | **Median \|rank shift\| triad-max** |
| --- | --- | --- | --- | --- | --- | --- | --- |
| Patient_27 | AD | 79 | 1.000 | 0.820 | 0.621 | 6.0 | 11.0 |
| Patient_53 | AD | 107 | 1.000 | 0.576 | 0.022 | 16.0 | 23.0 |
| Patient_95 | AD | 99 | 1.000 | 0.908 | 0.846 | 6.0 | 8.0 |
| Patient_12 | Control | 123 | 1.000 | 0.351 | 0.282 | 17.0 | 21.0 |
| Patient_90 | Control | 102 | 1.000 | 0.476 | 0.438 | 13.0 | 17.5 |
| Patient_humAD-86 | Control | 146 | 1.000 | 0.711 | 0.535 | 17.0 | 27.5 |
| Patient_94 | AD | 47 | 1.000 | 0.458 | 0.415 | 7.0 | 12.0 |
| Patient_22 | AD | 36 | 1.000 | 0.849 | 0.562 | 3.0 | 7.0 |
| Patient_111 | AD | 81 | 1.000 | 0.412 | 0.271 | 19.0 | 19.0 |
| Patient_107 | Control | 71 | 1.000 | 0.941 | 0.733 | 4.0 | 9.0 |
| Patient_101 | Control | 31 | 1.000 | 0.362 | -0.041 | 9.0 | 11.0 |
| Patient_28 | Control | 55 | 1.000 | 0.956 | 0.663 | 3.0 | 6.0 |

**E. Patient-level overlap of high-ranking LR sets**

| **Patient** | **Condition** | **Top-10 mean** | **Top-10 max** | **Top-20 mean** | **Top-20 max** | **Top-50 mean** | **Top-50 max** |
| --- | --- | --- | --- | --- | --- | --- | --- |
| Patient_27 | AD | 50.0% | 20.0% | 80.0% | 60.0% | 82.0% | 84.0% |
| Patient_53 | AD | 10.0% | 0.0% | 15.0% | 10.0% | 74.0% | 50.0% |
| Patient_95 | AD | 60.0% | 50.0% | 95.0% | 75.0% | 82.0% | 88.0% |
| Patient_12 | Control | 10.0% | 0.0% | 40.0% | 50.0% | 64.0% | 58.0% |
| Patient_90 | Control | 50.0% | 40.0% | 60.0% | 45.0% | 70.0% | 60.0% |
| Patient_humAD-86 | Control | 70.0% | 30.0% | 60.0% | 45.0% | 56.0% | 54.0% |
| Patient_94 | AD | 10.0% | 0.0% | 60.0% | 60.0% | 100.0% | 100.0% |
| Patient_22 | AD | 50.0% | 50.0% | 100.0% | 65.0% | 100.0% | 100.0% |
| Patient_111 | AD | 0.0% | 0.0% | 25.0% | 30.0% | 80.0% | 76.0% |
| Patient_107 | Control | 80.0% | 50.0% | 95.0% | 65.0% | 96.0% | 90.0% |
| Patient_101 | Control | 70.0% | 40.0% | 60.0% | 60.0% | 100.0% | 100.0% |
| Patient_28 | Control | 80.0% | 60.0% | 85.0% | 60.0% | 98.0% | 100.0% |

**Note.** All deltas are masked minus standard. A lower within-/between-cell-type distance ratio is favorable; higher cosine silhouette and kNN purity are favorable. The embedding analysis used matched cells in each patient, up to 200,000 sampled within-/between-type pairs, and up to 2,000 cells for cosine silhouette. The false-negative-aware sensitivity run used the same-cell-type and feature-similarity (>0.85) masking criteria while otherwise retaining the matched patient-specific inputs and training setup. For downstream analysis, triad-mean aggregates evidence across retained sender-LR-receiver triads, whereas triad-max is determined by the strongest retained triad. Spearman correlations used all evaluable LR pairs shared by the standard and masked models in each patient without an additional top-k cutoff. Top-k overlap is the shared fraction of the two top-k lists; when fewer than k LR pairs were evaluable, the effective k equaled the number available. The identical LR-set Jaccard values show that masking changed prioritization rather than candidate availability. Because improvements were patient- and metric-dependent rather than uniform, the masked objective is reported as a sensitivity analysis and the original InfoNCE formulation remains the primary model.
