# Supplementary material for: Spatial cell-cell communication inference based on cell-spot-ligand-receptor heterogeneous graphs
Source: PLoS Comput Biol. 2026 Sep 28;22(9):e1014835. doi: 10.1371/journal.pcbi.1014835 (PMC13674867; doi:10.1371/journal.pcbi.1014835)
Supplement: Supplementary file 14 [file pcbi.1014835.s014.docx]

**S10 Table. No-finetuning ablation analysis of SpaHCC ligand–receptor prioritization in Patient 27.**

This table compares ligand–receptor (LR) prioritization between the full SpaHCC model and the no-finetuning ablation. Agreement was evaluated using top-k overlap, Jaccard index, and score concordance across common LR pairs.

**Part A. Top-k LR priority overlap**

| **Comparison** | **Full SpaHCC** | **No-finetuning** | **Shared LR pairs** | **Jaccard index** | **Interpretation** |
| --- | --- | --- | --- | --- | --- |
| Ranked LR universe | 79 ranked LR pairs | 79 ranked LR pairs | 79 / 79 | 1.000 | Both models retained the same LR-pair universe. |
| Top-10 LR overlap | Top 10 | Top 10 | 0 / 10 | 0.000 | Highest-ranked LR priorities were not shared. |
| Top-20 LR overlap | Top 20 | Top 20 | 2 / 20 | 0.053 | Limited overlap among high-ranked LR candidates. |
| Top-50 LR overlap | Top 50 | Top 50 | 29 / 50 | 0.409 | Partial recovery of broader high-ranked LR candidates. |

**Part B. Score concordance across the 79 common LR pairs**

| **Score summary** | **Common LR pairs** | **Spearman rho** | **Pearson r** | **Interpretation** |
| --- | --- | --- | --- | --- |
| triad_max | 79 | -0.190 | -0.121 | Weak to negative concordance between full SpaHCC and no-finetuning scores. |
| triad_mean | 79 | 0.089 | 0.229 | Weak score concordance between full SpaHCC and no-finetuning scores. |

Note. The full SpaHCC and no-finetuning models were compared on the same 79 LR pairs. Top-k overlap indicates the number of LR pairs shared between the corresponding top-k lists. Correlation metrics were calculated across the 79 common LR pairs. Because raw triad scores differed in scale between models, the primary interpretation focuses on top-k overlap and rank/score concordance rather than direct score magnitude.
