# Supplementary material for: Spatial cell-cell communication inference based on cell-spot-ligand-receptor heterogeneous graphs
Source: PLoS Comput Biol. 2026 Sep 28;22(9):e1014835. doi: 10.1371/journal.pcbi.1014835 (PMC13674867; doi:10.1371/journal.pcbi.1014835)
Supplement: Supplementary file 15 [file pcbi.1014835.s015.docx]

**S11 Table. Sample-level AUROC values for the SpaHCC spatial ablation.**

The table reports AUROC values for 21 simulated benchmark samples for the pure non-spatial and full SpaHCC variants. Samples are numbered sequentially as Sample 1–Sample 21; no aggregate or additional performance metrics are included.

| **Sample** | **Pure non-spatial SpaHCC AUROC** | **Full SpaHCC AUROC** |
| --- | --- | --- |
| Sample 1 | 0.594398 | 0.499662 |
| Sample 2 | 0.661876 | 0.532319 |
| Sample 3 | 0.733761 | 0.526081 |
| Sample 4 | 0.708978 | 0.521002 |
| Sample 5 | 0.849249 | 0.512867 |
| Sample 6 | 0.753380 | 0.531367 |
| Sample 7 | 0.545185 | 0.521253 |
| Sample 8 | 0.587168 | 0.523323 |
| Sample 9 | 0.587154 | 0.512992 |
| Sample 10 | 0.649112 | 0.518986 |
| Sample 11 | 0.648795 | 0.525588 |
| Sample 12 | 0.618807 | 0.526731 |
| Sample 13 | 0.670716 | 0.523929 |
| Sample 14 | 0.692214 | 0.517819 |
| Sample 15 | 0.621357 | 0.526957 |
| Sample 16 | 0.607586 | 0.520320 |
| Sample 17 | 0.522756 | 0.525099 |
| Sample 18 | 0.597928 | 0.517078 |
| Sample 19 | 0.976680 | 0.527702 |
| Sample 20 | 0.984010 | 0.532354 |
| Sample 21 | 0.978171 | 0.528172 |
