# Supplementary material for: Spatial cell-cell communication inference based on cell-spot-ligand-receptor heterogeneous graphs
Source: PLoS Comput Biol. 2026 Sep 28;22(9):e1014835. doi: 10.1371/journal.pcbi.1014835 (PMC13674867; doi:10.1371/journal.pcbi.1014835)
Supplement: Supplementary file 16 [file pcbi.1014835.s016.docx]

**S12 Table. Directional resolution, cross-patient recurrence, receiver-state context, and CellChat provenance.**

This table summarizes fixed-LR directional support, cross-patient recurrence, exploratory receiver-state context, and the relationship between the scRNA-seq-derived CellChat prior source and final SpaHCC ranking.

**A. Fixed-LR conservative directional support**

| **Method** | **Median within-LR support percentile** | **Fraction with ligand and receptor AUC > 0.5** |
| --- | --- | --- |
| SpaHCC | 0.891 | 73.3% |
| COMMOT | 0.711 | 40.0% |
| CellNEST | 0.650 | 14.3% |
| Giotto | 0.614 | 28.6% |
| CellChat v2 | 0.888 | 69.2% |

*This is internal directional-resolution support because the scRNA-seq data contribute to SpaHCC construction; it is not independent accuracy validation.*

**B. Cross-patient recurrence of the EX→EX NRXN–NLGN module**

| **Module** | **Exact interactions** | **Sections** | **Event–section observations** | **SpaHCC median percentile** | **Comparator median percentile** | **Median difference** | **Positive sections** |
| --- | --- | --- | --- | --- | --- | --- | --- |
| EX→EX NRXN–NLGN | 3 | 4 | 12 | 0.997 | 0.355 | +0.641 | 4/4 |

**C. Exploratory EX→EFNA5–EPHA4→EX receiver-state context**

| **Section** | **Patient** | **Spearman ρ** | **BH FDR** | **Within-context \|ρ\| percentile** | **FDR < 0.05** |
| --- | --- | --- | --- | --- | --- |
| S1 | Patient_27 | 0.070 | 0.122 | 0.474 | No |
| S2 | Patient_53 | 0.084 | 0.0144 | 0.872 | Yes |
| S3 | Patient_95 | 0.171 | 1.45e-10 | 0.833 | Yes |
| S5 | Patient_111 | 0.200 | 7.99e-07 | 0.904 | Yes |

*The receiver-state analysis is exploratory and non-causal; it does not independently validate the inferred communication event.*

**D. scRNA-seq CellChat prior-source relationship with final SpaHCC ranking**

| **Section** | **Patient** | **Matched events** | **Spearman ρ** | **Fraction shifted ≥ 0.20 percentile** |
| --- | --- | --- | --- | --- |
| S1 | Patient_27 | 1400 | 0.390 | 27.5% |
| S2 | Patient_53 | 1500 | 0.387 | 29.7% |
| S3 | Patient_95 | 1500 | 0.747 | 5.8% |
| S4 | Patient_22 | 320 | 0.433 | 27.2% |
| S5 | Patient_111 | 832 | 0.639 | 23.3% |

*This prior-source analysis is distinct from the independently run ST-derived, cell-type-projected CellChat v2 comparator and is not independent validation.*
